# Understanding Insulin Endocrinology in Decapod *Crustacea*: Molecular Modelling Characterization of an Insulin-Binding Protein and Insulin-Like Peptides in the Eastern Spiny Lobster, *Sagmariasus verreauxi*

**DOI:** 10.3390/ijms18091832

**Published:** 2017-08-23

**Authors:** Jennifer C. Chandler, Neha S. Gandhi, Ricardo L. Mancera, Greg Smith, Abigail Elizur, Tomer Ventura

**Affiliations:** 1 GenEcology Research Centre, Faculty of Science, Health, Education and Engineering, University of the Sunshine Coast, 4 Locked Bag, Maroochydore, Queensland 4556, Australia; aelizur@usc.edu.au; 2 School of Mathematical Sciences, Queensland University of Technology, 2 George Street, Brisbane, Queensland 4000, Australia; neha.gandhi@qut.edu.au; 3 School of Biomedical Sciences, Curtin Health Innovation Research Institute and Curtin Institute for Computation, Curtin University, GPO Box U1987, Perth, Western Australia 6845, Australia; r.mancera@curtin.edu.au; 4 Fisheries and Aquaculture Centre, Institute for Marine and Antarctic Studies (IMAS), University of Tasmania, Private Bag 49, Hobart, Tasmania 7001, Australia; gregory.smith@utas.edu.au

**Keywords:** insulin-like growth factor binding protein (IGFBP), insulin-like androgenic gland peptide (IAG), insulin-like peptides (ILP1, ILP2), molecular modelling, binding interaction, alanine scanning, hotspot residue, electrostatics, decapod

## Abstract

The insulin signalling system is one of the most conserved endocrine systems of *Animalia* from mollusc to man. In decapod *Crustacea*, such as the Eastern spiny lobster, *Sagmariasus verreauxi* (Sv) and the red-claw crayfish, *Cherax quadricarinatus* (Cq), insulin endocrinology governs male sexual differentiation through the action of a male-specific, insulin-like androgenic gland peptide (IAG). To understand the bioactivity of IAG it is necessary to consider its bio-regulators such as the insulin-like growth factor binding protein (IGFBP). This work has employed various molecular modelling approaches to represent *S. verreauxi* IGFBP and IAG, along with additional Sv-ILP ligands, in order to characterise their binding interactions. Firstly, we present Sv- and Cq-ILP2: neuroendocrine factors that share closest homology with *Drosophila* ILP8 (Dilp8). We then describe the binding interaction of the N-terminal domain of Sv-IGFBP and each ILP through a synergy of computational analyses. In-depth interaction mapping and computational alanine scanning of IGFBP_N’ highlight the conserved involvement of the hotspot residues Q_67_, G_70_, D_71_, S_72_, G_91_, G_92_, T_93_ and D_94_. The significance of the negatively charged residues D_71_ and D_94_ was then further exemplified by structural electrostatics. The functional importance of the negative surface charge of IGFBP is exemplified in the complementary electropositive charge on the reciprocal binding interface of all three ILP ligands. When examined, this electrostatic complementarity is the inverse of vertebrate homologues; such physicochemical divergences elucidate towards ligand-binding specificity between Phyla.

## 1. Introduction

The binding interaction of insulin-like growth factor binding proteins (IGFBPs) and their insulin-like growth factor (IGF) ligands has been a significant focus of IGF endocrinology for the past two decades [1,2,3]. This is reflective of the central function of the high-affinity IGFBP subgroup (IGFBP1-6) in mediating the bioavailability and activity of IGFI and II at their receptor(s) [1,2,3]. In doing so, IGFBPs not only facilitate the translocation of their binding partners but they also provide proteolytic protection, extending the half-life and maintaining a functionally viable reservoir of the hormone in circulation [1,2,3].

The structure of the IGFBP is central to this function. Although domain specifics of the superfamily vary, most notably across the low-affinity IGFBP-related subgroup (IGFBP-rP1-9) [1], the family conforms to a common architecture: a highly structured, globular N terminal (N’) insulin-binding domain; a flexible linking domain; and a flexi-folded C terminal (C’) domain, which is the most variable domain across subgroups and species [1,3]. The highly structured N’ insulin-binding domain (the only domain conserved across the entire IGFBP superfamily) provides the primary binding interface for the ligand and is capable of binding in isolation [4]. In the case of IGFBP1-6, the C’ domain functions to maximise binding affinity, encapsulating the ligand to stabilise binding by interacting with the N’ domain [3,5,6]. In doing so, the C’ shields some of the key residues involved in the interaction of IGF with its receptor, increasing the antagonistic action of the IGFBP [5]. This synergistic binding of N’ and C’ domains is coordinated through the flexible linking domain [1].

We have identified an IGFBP homologue in the decapod crustacean *Sagmariasus verreauxi*, commonly referred to as the Eastern spiny lobster [7], prior to which a similar protein was identified in the red-claw crayfish (*Cherax quadricarinatus*) [8]. Additional homologues have since been found in a prawn, *Macrobrachium nipponense* [9], and two crab species, *Scylla paramamosain* [10] and *Callinectes sapidus* [11]. These decapod IGFBPs share closest homology with the human IGFBP-rP1 (known as MAC25) from the low-affinity IGF-binding subgroup. They all share a kazal-type serine proteinase inhibitor as the linking domain and an immunoglobulin-like domain as the C’ domain (rather than the thyroglobulin-type I domain of human IGFBP1-6 [1]). Unlike IGFBP1-6, the binding capacity of IGFBP-rP1 is more diverse, enabling it to bind insulin with a similar affinity as the IGFs, although with a reduced affinity compared to its specialised (IGFBP1-6) counterparts [12]. This is thought to be achieved through the substituted C’ immunoglobulin domain [1], which is proposed to reduce the synergistic N’ and C’ domain high-affinity binding for IGFs, whilst also better exposing the insulin binding site [13,14]. In addition, it has been recognised that although they share the same overall fold, the N’ insulin-binding domain of this IGFBP-r subgroup contains notable structural variations from IGFBP1-6, resulting in a decreased IGF binding affinity [15]. Thus some suggest that the IGF binding of the IGFBP-r subgroup is biologically irrelevant [15,16], advising of a primary function unrelated to IGF binding [17]. Even so, the general consensus appears to be that the IGFBP-r subgroup functions in both IGF-dependent and independent roles [1,17].

In the context of the decapod IGFBPs, the homology with the less IGF-specific IGFBP-rP1 is likely to have functional significance, relating to the ligands with which these decapod homologues bind. The IGFs comprise one subgroup of the insulin-like superfamily, with the insulin-like peptides (ILP) encompassing the other [7,18]. The structural distinction centres around the pre-prohormone structure and processing. IGFs tend to retain their truncated C-domain and have additional D (uncleaved) and E (cleaved) domains after the A-chain [2,19,20]. ILPs undergo cleavage of the C-peptide and terminate after the A-chain [7,18]. However, both IGFs and ILPs share the same disulfide bond topology, with two inter (B to A) and one intra (A)-chain bonds [18,20].

IGF homologues have not yet been identified in decapods, but the Crustacean class *Malocastraca* (which includes the Order *Decapoda*) is known for an ILP termed the insulin-like androgenic gland peptide (IAG). This hormone, only found in males (with noted exceptions [21,22]), is specifically produced and secreted from a male-specific endocrine gland known as the androgenic gland (AG) [23,24,25]. Upon secretion, IAG stimulates and maintains the broad tissue effects of male sexual differentiation and maturation [26,27,28,29,30], reviewed in [31]. More recently, the prevalence of ILPs in these species has diversified with the first identification of a DILP7/relaxin-like ILP in *S. verreauxi* (Sv-ILP1) [7], since identified across the Order [32].

Work in *C. quadricarinatus* has already demonstrated the capacity of Cq-IGFBP to bind Cq-IAG through a pull-down assay with AG homogenate, where the IGFBP was shown to bind residues within the A-chain, B-chain and C-peptide; highlighting the ability of the IGFBP to also bind the IAG pre-prohormone [8]. The IGF/ILP receptor signalling system, as characterised in mammals [2,3] and *Drosophila* [33], is conserved in decapods (as evidenced by the identification of an active tyrosine kinase insulin receptor (TKIR) [34,35] and an inactive decoy (TKIR_decoy) [34]). Consequently, it seems highly likely that the IGFBP will adopt a similarly conserved role within the system. Thus, to realistically interpret the bioactivity of IAG in mediating male sexual development, we must integrate the regulatory influences of the IGFBP. Furthermore, the identification of additional ILPs [7] and the broad tissue distribution of the IGFBP [7,8,10,11] in the decapods may suggest a multi-ligand binding role.

In light of the dramatic advancements that have been made in the field of computational protein-modelling and interaction studies [36,37], this work employed an in silico approach to study the IGFBP_N’-ILP ligand interaction in decapod *Crustacea.* Firstly, we present Sv-ILP2 and Cq-ILP2, which are novel to the Order. We then model the N’ domain of Sv-IGFBP and each ligand (Sv-IAG, ILP1 and ILP2) in order to characterise the binding interaction of each. In doing so we determine a subset of consistently interacting residues at the IGFBP interface, involved in binding all three ligands, which are further suggested to be hotspots based on computational alanine scanning. Electrostatic potential surface calculations illustrate the significance of the negatively charged hotspots, suggesting them to be a fundamental feature of complex formation. Together, these analyses emphasise the consequence of amino-acid variations in determining the physicochemical structure and consequential binding interactions of the seemingly conserved N’ insulin-binding domain of the IGFBP.

## 2. Results

### 2.1. Identification of Sv-ILP2

**Figure 1:**
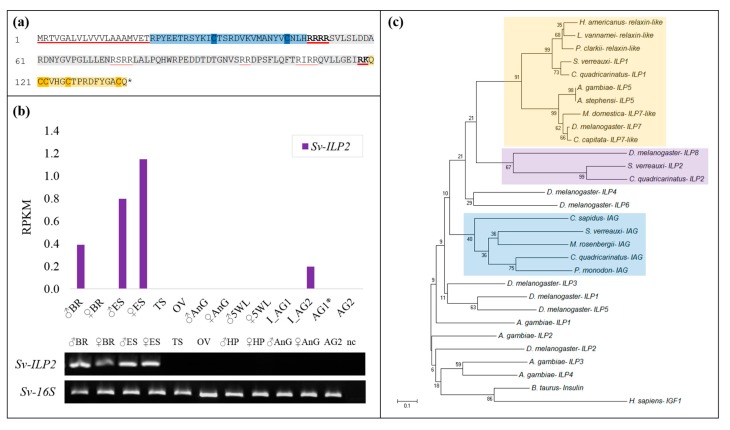
Spatial expression and phylogeny of Sv-ILP2: (**a**) sequence of Sv-ILP2, the signal peptide is underlined in red and the B-(blue) and A-(orange) chains boxed with the cysteine core of each highlighted. The C-peptide is shown in grey with the predicted Arg-C cleavage sites shown, with those predicted to generate the mature hormone underlined in red. (**b**) Transcriptomic spatial expression of *Sv-ILP2* quantified as reads per kilobase per million reads (RPKM) for male and female brain (BR), eyestalk (ES), gonads (TS and OV), antennal gland (AnG), and fifth walking leg (5WL), immature androgenic glands (I_AG1 and I_AG2), and mature androgenic glands (AG1* and AG2, where * indicates a hypertrophied gland). Validated with spatial RT-PCR analyses, with the removal of the immature AGs, 5WL, and AG1*, and the addition of male and female hepatopancreas (HP). Negative control (NC) in the fifteenth lane, *16S* as positive control. (**c**) Neighbour-joining phylogram of Sv- and Cq-ILP1 and ILP2 with a range of ILPs from model species: *Anopheles* ILP1-5, *Drosophila* Dilp1-8, decapod insulin-like androgenic gland peptides (IAGs), and bovine insulin and human IGFI. Bootstrap values are shown at each node and were performed with 1000 replicates. Scale bar indicates number of amino acid substitutions per site. IAG cluster boxed in blue, Dilp7/relaxin-like cluster in yellow, and novel decapod ILP2s in purple.

This work is the first to describe the identification of a second insulin-like peptide in *S. verreauxi*, Sv-ILP2 (KP006646). Sv-ILP2 conforms to the ILP superfamily structure exhibiting all of its conserved features: a signal peptide (20 amino acids (aa)); followed by a B-chain (28 aa) containing two cysteines; a C-peptide (71 aa), flanked by RR cleavage sites; and an A-chain (17 aa) containing a double and two single cysteines (Figure 1a). Interestingly, unlike Sv-IAG and Sv-ILP1, Sv-ILP2 contains multiple RR cleavage sites throughout the C-peptide. These additional chains are somewhat reminiscent of the additional D and E domains of the IGF-like pre-prohormone structure. However, as the CCxxxC cysteine signature is located in the C’ domain of the open reading frame it was considered to be the A-chain, suggesting these additional cleavage sites constitute an elongated C-peptide; we therefore classified Sv-ILP2 as an ILP rather than an IGF.

Spatial expression analyses of *Sv-ILP2* (Figure 1b) show it to be predominantly expressed in the neuroendocrine tissues (brain and eyestalk) of males and females, although all RPKMs are low (RPKMs as follows: female eyestalk 1.15; male eyestalk 0.8; male brain 0.39; immature AG 0.2). Temporal RT-PCR analyses were also conducted (from phyllosoma instar 16 to puerulus) but *Sv-ILP2* expression was not identified (data not shown). When blasted at NCBI (https://blast.ncbi.nlm.nih.gov/Blast.cgi), neither the pre-pro nor mature hormone gave any significant hits, although our phylogenetic analyses (with a range of model ILPs) shows that Sv-ILP2 clusters with Dilp8 (Figure 1c). Thus, Sv-ILP2 is the first of a new subclass of ILPs to be described in the decapods.

This work also presents two homologues of Sv-ILP1 and ILP2 (*S. verreauxi*, Suborder *Achelata*) in the red-claw crayfish, *C. quadricarinatus* (Suborder *Astacidea*); we have therefore named these peptides Cq-ILP1 (KP006644) and Cq-ILP2 (KP006645) (Figure 1c). Cq-ILP1 is comprised of: a signal peptide (25 amino acids (aa)); a B-chain (37 aa); a C peptide (115 aa); and an A-chain (37 aa). Cq-ILP2 is comprised of a: signal peptide (25 aa); a B-chain (29 aa); a C-peptide (159 aa); and an A-chain (17 aa). When assessed by RT-PCR, the Cq homologues display similar spatial expression to that described in *S. verreauxi*, with *Cq-ILP1* present in the male and female brain, antennal gland, gonads, and the female, but not male hepatopancreas, while *Cq-ILP2* expression is specific to the male and female brain and thoracic ganglia (no expression in the eyestalk) (data not shown).

### 2.2. Sequence Analyses of IGFBPs and ILP Ligands

**Figure 2:**
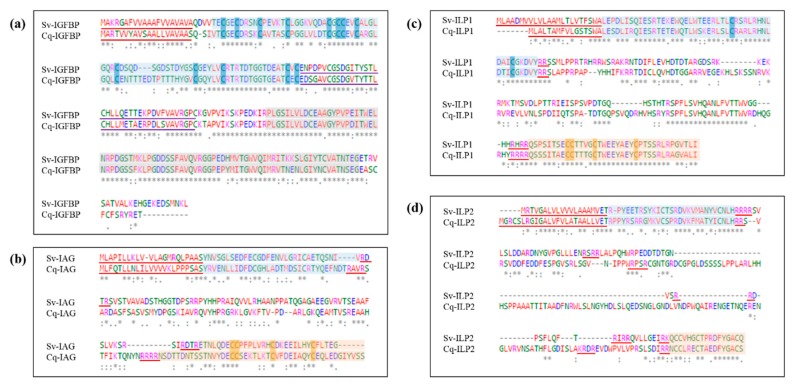
Sequence alignment of *Sagmariasus verreauxi* and *Cherax quadricarinatus* IGFBP and ILP homologues, with emphasis on the conservation of physicochemical properties. Residues are colored in accordance with properties: red—defines small, hydrophobic residues; blue—negatively charged/acidic; magenta—positively charged/basic; and green—polar and amine groups. An asterisk (*) indicates a conserved amino acid, a colon (:) those with conserved physicochemical properties, and a full stop (.) those with weakly similar properties. (**a**) Compares Cq-IGFBP and Sv-IGFBP, the signal peptide is underlined in red, the insulin-binding domain boxed in blue with the cysteine core highlighted, the kazal domain underlined in purple, and the C’ immunoglobulin domain boxed in grey. (**b**) Compares Cq-IAG and Sv-IAG; (**c**) Sv-ILP1 and the novel Cq-ILP1; and (**d**) the novel Sv-ILP2 and Cq-ILP2. In each case the signal peptide is underlined in red and the mature hormone is highlighted as the B-(blue) and A-(orange) chains with the cysteine core of each highlighted; C-peptide Arg-C proteinase cleavage sites are underlined in red.

We conducted pairwise alignment of the IGFBP, IAG, ILP1, and ILP2 peptides from *S. verreauxi* and *C. quadricarinatus* to assess physicochemical conservation. The IGFBP sequences share a pairwise identity score of 68.9% and significant conservation in physicochemical properties (Figure 2a). With regard to the ligands, the IAG peptides share the lowest identity score of the three ILPs, at 32.2% across the pre-prohormone and 35.5% across the mature hormone (consisting of only the A and B-chains) (Figure 2b). The ILP1 homologues share the highest conservation at 64.6% across the pre-prohormone and 83.1% across the mature hormone (Figure 2c). This high identity score is fitting with the strong conservation of this relaxin-like ILP subclass across species (also known as the Dilp7-likes characterized in *Drosophila*).

The ILP2 homologues share an identity score of 44.6% at the pre-prohormone level, which increases to 57.8% for the mature peptide (Figure 2d). As previously mentioned, we surmise that the final cleavage site indicates the beginning of the A-chain, based on the structural placement of the cysteines but also reflective of the increased conservation observed between the Sv- and Cq-ILP2 homologues in this C’ domain. These sequence alignments exemplify the minimized evolutionary restraint within the C-peptide, showing far higher rates of divergence than that observed in the A and B-chains which ultimately form the mature and active hormone.

**Figure 3:**
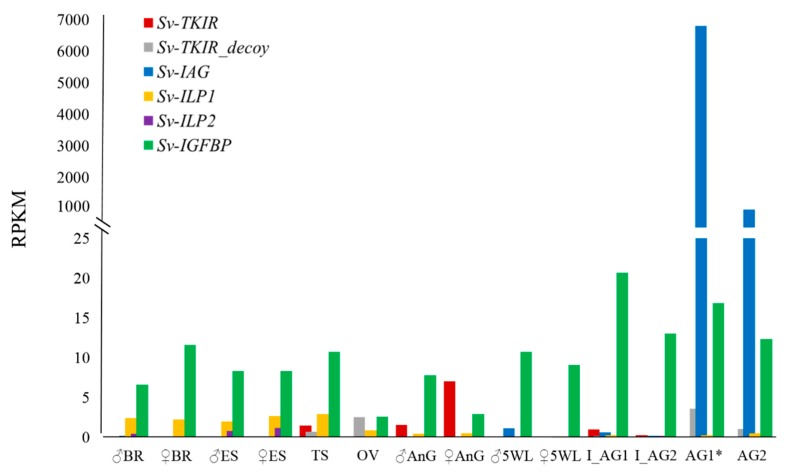
Spatial expression profile summarising all insulin factors identified in *Sagmariasus verreauxi* for a cohesive depiction of our current understanding of the insulin endocrine system: IAG [38]; ILP1 and IGFBP [7]; the active (TKIR) and decoy (TKIR_decoy) tyrosine kinase insulin receptors [34,39]; and Sv-ILP2. Quantified as RPKM; tissue abbreviations as previously described.

To provide a more cohesive understanding of the insulin-signaling system in *S. verreauxi*, we generated a spatial expression profile summarizing all of the insulin factors identified in the species to date (Figure 3), namely, Sv-IAG [38]; Sv-IGFBP and Sv-ILP1 [7]; Sv-TKIR and Sv-TKIR_decoy [34,39]; and Sv-ILP2, presented in this work (all RPKMs have been validated with independent RT-PCR, some with additional in situ analyses; related references given above after each gene). Of note is the broad tissue distribution of Sv-IGFBP and the dramatically higher expression of IAG relative to all other endocrine factors. However, this expression must be considered in the context of localization, as IAG is secreted by a relatively small subset of cells, all of which are accounted for in this one gland.

### 2.3. Structural Modelling

**Figure 4:**
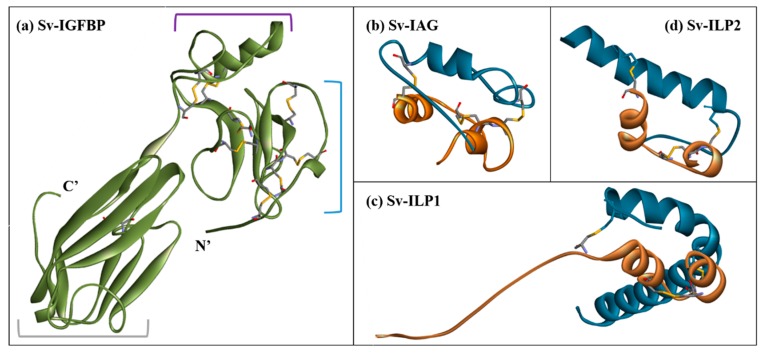
Molecular structure models of (**a**) Sv-IGFBP, the domains of which are indicated with: a blue bracket for the highly structured N’ insulin-binding domain; a purple bracket for the linking kazal domain; and grey bracket for the C’ immunoglobulin domain. (**b**) Sv-IAG, (**c**) Sv-ILP1, and (**d**) Sv-ILP2 are shown as previously described with the A-chain in orange and B-chain in blue. All structures are shown in secondary structure ribbon format with disulfide bonds highlighted as sticks.

Structures of Sv-IGFBP (3TJQ and 2CQV) and Sv-IAG, Sv-ILP1 and Sv-ILP2 (2KQP) were predicted by homology modelling using the described PDB templates. The predicted structure of Sv-IGFBP provides clear visualization of the domain architecture of the molecule (Figure 4a). The highly structured N’ insulin-binding domain contains seven disulfide bonds, ensuring accurate folding of the binding interface. The kazal domain, defined as the flexible linking region which connects the N’ and C’ domains, contains an alpha helical region and one disulfide bond. The C’ immunoglobulin domain also contains a disulfide bond, as well as a *cis*-peptide bond, but lacks any other significant orienting features and is mainly comprised of beta pleated sheets and random coils.

Each ILP ligand conforms to the characteristic tertiary structure of the ILP family with two inter and one intra (A-chain) disulfide bonds that determine the overall fold of the molecule (Figure 4b–d). Within the confines of the generic ILP fold, each protein displays unique features. The most prominent of these include the elongated A-chain of Sv-ILP1, which does not form the usual alpha-helix but instead a random coil (Figure 4c; Figure S1). Conversely, Sv-ILP2 has a truncated A-chain, where the residues _1_QCCV_4_ result in an alpha turn rather than a complete helix (Figure 4d; Figure S1). Refer to M&M and Figure S1 for full modelling procedures. It is these features that determine the specifics of the interactive interface shared between the A- and B-chains, dictating the molecular structure of each ligand. This is best illustrated by the inter-chain interactions: the A- and B-chains of IAG, ILP1, and ILP2 are predicted to share one, three, and one hydrogen bonds and 153, 109, and 74 non-bonded contacts, respectively, in addition to the two disulfide bonds common to all three (predicted by PDBsum) (Figure S2).

### 2.4. Complex Formation

**Figure 5:**
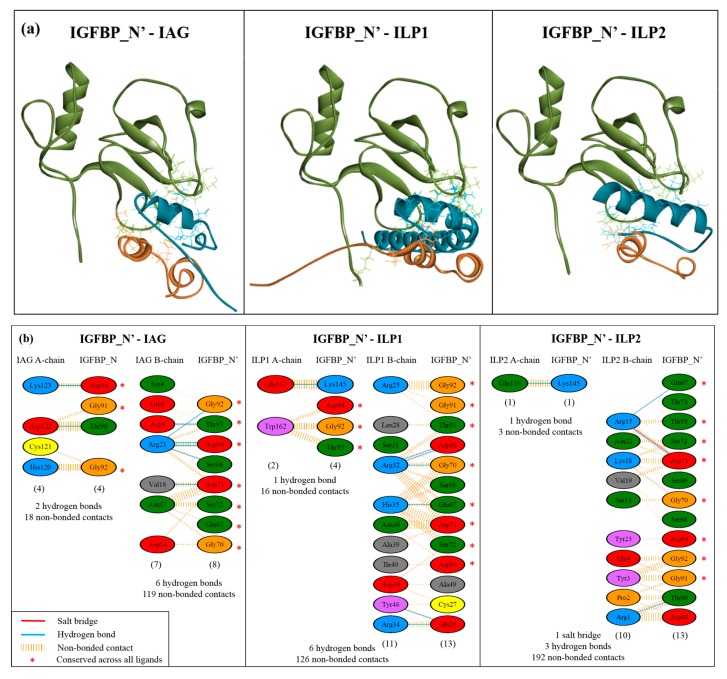
Molecular structure models of bound complexes and interaction map: (**a**) Complex of Sv-IGFBP_N’ with each ligand. In each case the IGFBP_N’ is in green and the ligand in orange (A-chain) and blue (B-chain). All residues involved at the binding interface are indicated in stick, with colouring as previously described. (**b**) Interaction map describing residue-specific interactions between Sv-IGFBP_N’ and each ligand. Standard amino acid abbreviations are used, with colours indicating physicochemical properties as follows: blue—positive, red—negative, green—neutral, grey—aliphatic, mauve—aromatic, orange—proline and glycine, and yellow—cysteine. Number of interacting residues is given in brackets; an asterisk denotes those residues that show a conserved binding interaction with all three ligands (*n* = 8).

Complex formation and protein-protein interactions were then investigated through both manual structural alignment and predictive binding analyses. Due to the reduced reliability of modelling the bound C’ immunoglobulin domain, Sv-IGFBP was truncated after the kazal domain (Sv-IGFBP_N’) (as is common in IGFBP binding studies [40,41]; PDB: 1H59 [42]; PDB: 1WQJ [5]). Sv-IGFBP_N’ was used for all further interaction studies. We collated manual alignment analyses, with predicted binding interactions generated through PDBsum [43] and PRODIGY [44] to generate an interaction map of all the residues involved in complex formation (Figure 5). Visual comparison of the three Sv-IGFBP_N’ complexes (Figure 5a) clearly demonstrates the binding interface of the N’ insulin-binding domain (supported by HADDOCK2.2 simulations: Figure S3), with all highlighted interacting residues predicted by both PDBsum and PRODIGY (shown in Figure 5b; additional residues predicted by PRODIGY presented in Table S1). Of all the predicted interacting residues presented in Figure 5b, we have highlighted those amino acids of IGFBP_N’ that show conserved interaction contacts with all three ligands (*), namely: the negatively charged Asp(D)_71_ and Asp(D)_94_; supported by the polarGln(Q)_67_ (where proton acceptor properties enable it to form two hydrogen bonds, stabilizing the overall negative charge); the neutrally charged Ser(S)_72_ and Thr(T)_93_; and Gly(G)_70_, Gly(G)_91,_ and Gly(G)_92_. In addition to these eight consistent contacts of IGFBP_N’, PRODIGY predicts a further nine (Table S1). The physicochemical nature of each interaction as predicted by PRODIGY suggests that a relatively even contribution of charged, polar, and non-polar interactions contribute to binding, with charged forces slightly dominating with IAG and ILP1 and hydrophobic forces being slightly dominant with ILP2 (Table S1b).

### 2.5. Interaction Hotspots

**Figure 6:**
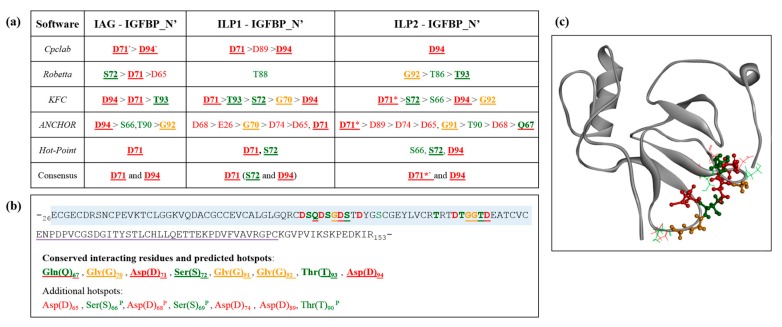
Identification of hotspot residues: (**a**) determined by five independent alanine scanning algorithms. Amino acids are shown in standard letter notation. Other notation is as follows: an asterisk (*) indicates the presence of a salt bridge predicted by PDBsum; a dash (`) a salt bridge predicted by Cpclab; bold underlined indicates residues that show a conserved binding interaction with all three ligands (see Figure 5b). (**b**) Illustration of Sv-IGFBP_N’ sequence (insulin-binding domain boxed in blue and kazal domain underlined in purple) highlighting the positioning of predicted hotspots. The eight consistently interacting hotspot residues are in bold underline. Additional predicted hotspots are also listed, with a ^P^ highlighting those residues that were predicted as consistently interacting residues by PRODIGY. (**c**) Structural illustration of Sv-IGFBP_N’ with conserved interacting hotspot residues highlighted in ball and stick and additional hotspots in stick. Throughout, red indicates negatively charged residues; green, neutrally charged; and orange, glycine. Note the neutral Gln(Q) has been underlined in red in (**b**) sequence and coloured red in (**c**) structure due to its role in stabilizing the overall negative charge through the formation of hydrogen bonds.

As the IGFBP_N’ is predicted to contain a subset of consistently interacting residues when in complex with all three ligands, the energy contributions of these residues were investigated using computational alanine scanning to highlight binding hotspots. A hotspot is defined as an amino acid that significantly decreases the binding free-energy of complex formation. As a rule: hotspot potential increases as the degree of buriedness of a residue decreases (due to shape complementarity); it increases if a given residue forms a salt bridge, and hotspot residues tend to show structural and interactive complementarity to that of their binding partners. In brief, alanine scanning is conducted by mutating each residue in the complex to alanine, removing the sidechains that are fundamental to its physicochemical and interaction properties. The binding free-energy is then calculated and compared to the wild-type. In doing so, those residues that contribute most significantly to achieving the binding energetics of complex formation are determined. This work used five independent algorithms to generate a meta-style analysis. These hotspot predictions are in strong support of our predicted interactions, with all the consistently interacting residues predicted by both PDBsum and PRODIGY being identified as hotspots (Figure 6a). Furthermore, of the additional predicted hotspots, four of the seven residues were predicted as consistently interacting residues by PRODIGY (denoted with a ^P^ in Table S1). Together, this allows confident prediction that these residues are vital to the binding capacity of IGFBP_N’, suggesting a synergistic physicochemical influence of negative charge, polar neutral residues, and glycine. Figure 6b shows the sequence positioning of these residues, which appear to cluster into two defined regions as hotspot pockets, which, at the structural level, orientate across the exposed binding interface (Figure 6c).

### 2.6. Electrostatic Potential Molecular Surfaces

**Figure 7:**
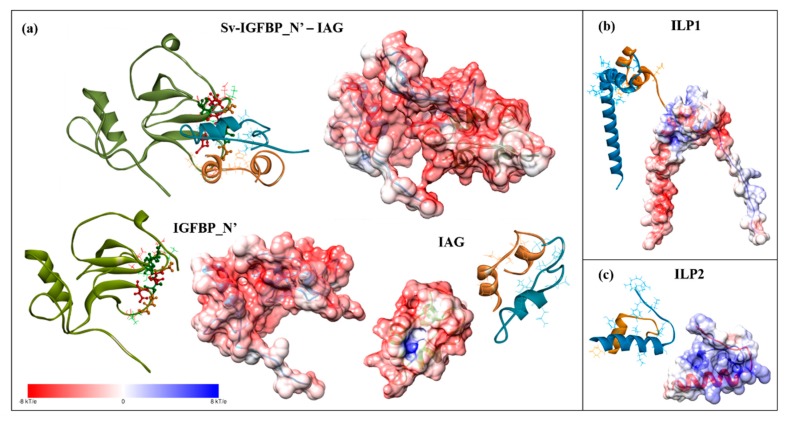
Electrostatic potential surface of: (**a**) Sv-IGFBP_N’-IAG complex and the individual binding partners; (**b**) ILP1; and (**c**) ILP2. IGFBP_N’ is coloured in green and the ligands in blue and orange; the interacting hotspot residues of IGFBP_N’ are highlighted as described in Figure 6c; ligands have been orientated to display the binding interface. Surfaces are colored by potential on the solvent accessible surface on a scale of −kT/e (red) to +kT/e (blue), as indicated by the scale bar.

**Figure 8:**
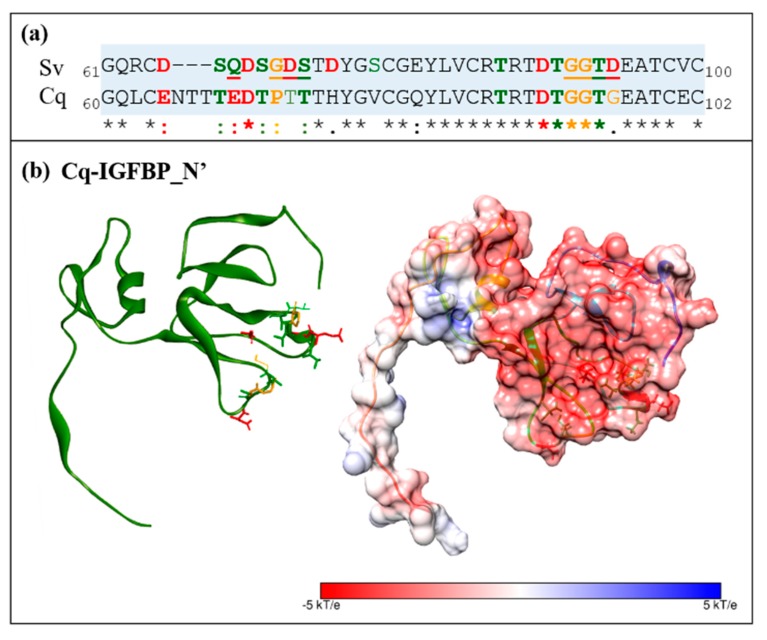
Comparison of Sv-IGFBP_N’ and Cq-IGFBP_N’: (**a**) sequence alignment of the focal region of Sv and Cq insulin-binding domains (boxed in blue); (**b**) Electrostatic potential surface of Cq-IGFBP_N’. All physicochemical conserved residues (predicted as interaction contacts in Sv) are highlighted in bold in sequence and as sticks in structure, the two non-conserved residues (Thr_75_ and Gly_97_) are shown in line. Throughout, red indicates negatively charged residues; green, neutrally charged; and orange, proline and glycine. Surfaces are colored by potential on the solvent accessible surface on a scale of −kT/e (red) to +kT/e (blue), as indicated by the scale bar.

Considering the conserved prevalence of negatively charged residues throughout the hotspot predictions, we conducted an electrostatic potential surface analysis to further investigate the significance of these negatively charged residues. Figure 7a shows the electrostatic potential surface of the IGFBP_N’-IAG complex and the binding interfaces of each individual molecule, as well as the additional ligands, ILP1 (Figure 7b) and ILP2 (Figure 7c). The binding interface of Sv-IGFBP_N’ is indeed characterized by a strong negative electrostatic potential. The complementary positive electrostatic potential that exists on the reciprocal interface of all three ligands is in support of an electrostatic interaction. Considering the sequence conservation of Sv-IGFBP and Cq-IGFBP (Figure 2a), we performed similar analyses on Cq-IGFBP_N’. Indeed, the negative electrostatic potential of the predicted binding interface is conserved to Cq-IGFBP_N’, as is the broader physicochemical nature of 13 of the 15 interaction hotspots characterized in Sv (Figure 8). Of note is the Glu(E)_70_ substitution in Cq (replacing the Gln(Q)_67_ of Sv) emphasizing the suggested negative-centered properties of this residue in Sv. This is indicative of conserved interactive properties and the resulting binding mechanism of the two IGFBP_N’ of these species.

**Figure 9:**
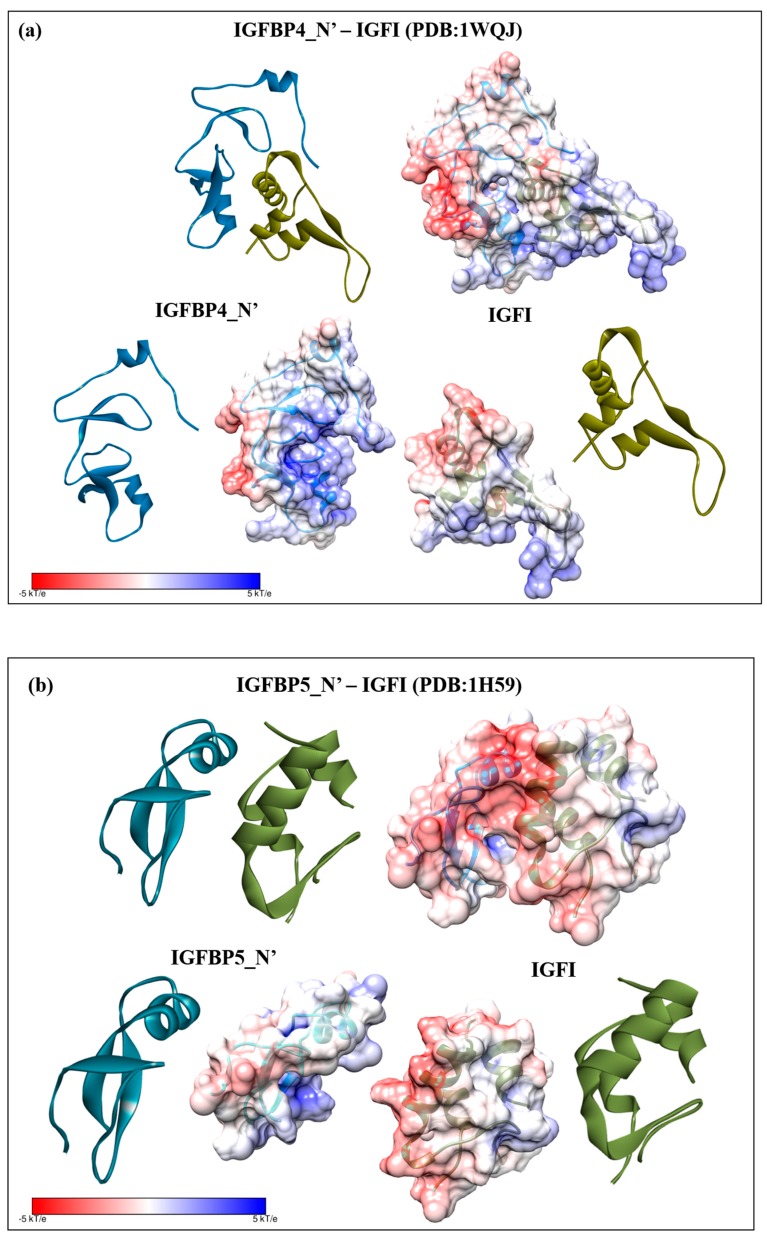
Electrostatic potential surface in vertebrates: (**a**) the bound complex and individual binding partners of human IGFBP4_N’-IGFI (PDB: 1WQJ); and (**b**) human IGFBP5_N’-IGFI (PDB: 1H59). In both cases the IGFBP_N’ is coloured in blue and the IGFI in green. Surfaces are colored by potential on the solvent accessible surface on a scale of −kT/e (red) to +kT/e (blue), as indicated by the scale bar.

For evolutionary comparison, we conducted electrostatic potential surface calculations on the template complexes IGFBP4_N’ and IGFI (PDB: 1WQJ) (Figure 9a), and IGFBP5_N’ and IGFI (PDB: 1H59) (Figure 9b), as well as IGFII (PDB: 2L29) (Figure S4), and found that these human complexes (positive electrostatic potential on the IGFBP and negative on the ligand) display inverse complementary electrostatic potentials to those described in *S. verreauxi* and *C. quadricarinatus* (negative electrostatic potential on the IGFBP and positive on the ligand). We then proceeded to conduct electrostatic potential surface calculations on a range of publicly available vertebrate IGFBP1_N’ and IGFI structures. All of those analyzed (namely rat, cow, chicken, for which IGFBP2_N’ replaced IGFBP1 and salmon) display a similar electrostatic potential complementarity to that observed in human, with a positive (IGFBP) and negative (IGF) electrostatic potential (data not shown). Thus, the complementarity common to the vertebrate IGFBP-ligand examples differs from that described in these decapod *Crustacea*.

## 3. Discussion

The interaction studies conducted in this work provide structural and physicochemical evidence for the capacity of IGFBP to bind the ILP ligands identified in *S. verreauxi*. By comparing Sv002D-IGFBP with a homologous IGFBP from *C. quadricarinatus* (a member of the sister Suborder *Astacidea*), we highlight the conservation of these physicochemical properties, suggesting a similar binding interaction to be conserved. These structural studies are indicative of a conserved function of the IGFBP in the insulin endocrine system of these decapods.

The contacts that occur at the binding interface are the fundamental features that dictate a binding interaction and, critically, its stability [45]. This makes in silico analyses such as these a highly suitable method to investigate and visualise molecular binding. Furthermore, the development of computational alanine substitution has provided significant insight into the energetic contributions of the binding interface [45,46], most significantly highlighting that only a few key residues—the hotspots—are those that contribute most significantly to the binding free-energy of complex formation [45]. Our analyses of Sv-IGFBP_N’ agree with this, firstly identifying those residues that are involved in interaction contacts with all three ligands (Figure 5b) and then verifying their significance as interaction hotspots. Hotspot residues tend to cluster in pockets within the centre of the exposed binding interface [45]. This is true of our predicted hotspots, which show close structural orientation across the exposed centre of the N’ domain (Figure 6c).

The physicochemical nature of these conserved interacting hotspots (Q_67_, G_70_, D_71_, S_72_, G_91_, G_92_, T_93_ and D_94_) suggests that a range of contact properties exist at the binding interface (supported by the PRODIGY contact predictions; Table S1b). In particular, we illustrate the significance of the negatively charged hotspots, providing a structural illustration of the negative charge of the entire Sv-IGFBP_N’ binding interface. Taken with the complementary positive charge of the reciprocal interface of each ILP (Figure 7), it appears that complex formation occurs, at least in part through an electrostatic interaction. Electrostatic interactions promote complex affinity through hydrogen bonding and in certain cases, such as that predicted for Asp(D)_71_ in complex with ILP2 (Figure 5b), salt bridge formation, adding significant stability to the bound complex.

However, any binding interface is achieved through a complex synergy of molecular interactions [45]. For example, hydrophobicity has been repeatedly described as the interactive force in IGFBP_N’-IGF complexes. Studies with human IGFBP5_N’ [42] and IGFBP4_N’ [6] highlight the conserved importance of the hydrophobic residues (Val_49/48_, Leu_70/69_, Leu_74/72_). This hydrophobic patch is conserved across all six IGFBP_N’ [47] and has been mutated in IGFBP3-6_N’, resulting in a ~1000 fold decrease in binding affinity [40,41,48,49,50]. The solved complex of IGFI with IGFBP5_N’ further verified the hydrophobic interaction, evident through the interwoven hydrophobic contacts of protruding side chains [42], also described for IGFBP4_N’ [5,6]. Of the above, the only mention of electrostatic properties comes from IGFBP3_N’ and 5_N’, where the electropositive residues Lys_68/_Arg_69_ were also highlighted as critical for high-affinity binding [40,41]. It is only more recently that the role of electrostatic interactions has been established. Chen et al., (2014) [51], conducted computational alanine scanning of IGFI to select mutation hotspots in order to conduct comparative molecular dynamic simulations. Five of the six determined hotspots were negatively charged (three Glu(E) and two Asp(D)) and electrostatic interactions were determined to be the dominant driving force behind the IGF-IGFBP interaction [51]. These simulations are in strong support of our electrostatic potential surface analyses, which describe an electropositive (IGFBP) to electronegative (IGF) complementarity in vertebrates (Figure 9 and Figure S4).

It follows that residues across the binding interface coevolve, acquiring binding pockets enriched with amino acids that ensure an interdependent binding interaction [45]. Such coevolution is evident in the significant sequence conservation between Sv and Cq-IGFBP, and further still by the inverse electrostatic complementarity that we have observed between the crustacean (Figure 7 and Figure 8) and vertebrate (Figure 9 and Figure S4) IGFBP_N’-ligand complexes. Rosen et al., (2013) [8], found that Cq-IGFBP was not able to bind human insulin and could only weakly bind human IGFI. This emphasises that although the conserved cysteine architecture of the IGFBP_N’ family coordinates the same overall fold [1,15], it is the specific properties of amino acids, particularly the interaction hotspots, that govern side-chain interactions [15] and thus the binding capacity for any given ligand. Indeed, the same has been noted with the evolutionary conservation of the insulin receptor and its interactions with insulin [52].

A structural comparison of the bound Sv-IGFBP_N’ complexes suggests that a similar interaction is shared across all three ligands. Binding affinity predictions varied, with PRODIGY [43,44] and PYDOCK [53] showing no significant distinction and ROSETTADOCK [54] indicating an ILP1 > IAG > ILP2 affinity pattern and FIREDOCK [55] an ILP2 > ILP1 > IAG pattern. Thus, no reliable affinity prediction can be determined; however, taken with our structural studies, it can be stated that Sv-IGFBP_N’ appears to lack a selective affinity for any of these ligands, similar to that described for IGFBP-rP1 with insulin, IGFI, and IGFII [12]. The inability to generate consistent predictions of relative binding affinity is partly due to the intrinsic error in the computational prediction of binding affinities but also reflects the use of a molecular model of the isolated IGFBP_N’. Indeed, this is a common problem in IGFBP structural studies. Although well aware of the interactive nature of the IGFBP_N’ and C’ domains in the mediation of binding affinity, the N’ insulin-binding domain remains the focus of structural and affinity studies, mainly due to the poor ability to solve the flexible linking domain [51]. Yet, as we are well aware of the synergistic function of the N’ and C’ domains in mediating affinity [3,6], we must strive to generate interaction studies of the entire protein [37] in order to gain accurate in silico quantification of IGFBP binding affinity across ligands.

In a practical context, these in silico proof-of-binding studies suggest that these decapod IGFBPs may offer a distinct mode to regulate the bioavailability and consequential activity of IAG. The use of *RNAi* biotechnologies employing IAG to induce sex-reversal for the monosex population culture has been highly successful in the commercial decapod *M. rosenbergii* [56,57], with similar research practices occurring across commercial species. Molecular evidence for the interconnected nature of the IGFBP and IAG was demonstrated by Li et al., (2015) [9], who showed that the silencing of *IAG* in *M. nipponense* caused a ~50% reduction in the expression of the *IGFBP* (so named *IAGBP*). However, although this is evidence of a transcriptional interaction, when interpreted in the context of this work these conclusions may be somewhat misleading. This is most evident in the naming of the IGFBP as an IAGBP, suggesting specificity. This study clearly demonstrates the capacity of the IGFBP to bind non-IAG ILPs. Indeed, the finding that *IAG* silencing only resulted in a significant decline of *IAGBP* in the AG, testis, muscle, and hepatopancreas, but not in the neuroendocrine tissues of brain, eyestalk, and nerve cord is evidence for a maintained function of the IAGBP in these tissues. As we show both *ILP1* [7] and *ILP2* (this study) to express in neuroendocrine tissues, perhaps the maintained expression of “*IAGBP*” in the neuroendocrine tissues of *M. nipponense* is evidence of an unaffected interaction with additional ILPs. This is further supported by the increasing evidence of additional ILP1/relaxin-like ILPs in the decapods [32], which are likely to share a similar binding interaction with their IGFBPs. Thus, we caution against employing IGFBP as a target for IAG manipulation, as it is likely to induce off-target effects across the broader insulin endocrinology of these species. In the context of IAG regulation, additional bio-regulators may provide a more specific anti-protease action, such as the family of AG enriched α2-macroglobulins [58] identified through their >5× higher expression in the AG relative to all other tissues [59].

Moreover, the IGF-independent action of the IGFBP superfamily (most significantly the IGFBP-r subgroup) is not to be ignored [3,17]. Thus, an ILP-independent functionality of these decapod IGFBPs is very probable; an example being the immunological function investigated by Huang et al., (2016) [11]. When one considers the unspecified IGF/insulin binding capacity of IGFBP-rP1, as well as its ligand independent functions [1], perhaps these homologous decapod IGFBPs [7,8,9,10,11] (which are the only IGFBP subtype to be identified in the Order) are the multi-functional, unspecified ancestors of the superfamily described in vertebrates. Multiple modes for the evolution of the IGFBP family have been suggested, but establishing the evolutionary trajectory of this diverse superfamily is complex, with the only pronounced feature being the early emergence and conservation of the N’ insulin-binding domain [1].

In summary, this work has added novel evolutionary perspectives to the IGFBP superfamily, demonstrating the conserved functionality of an IGFBP-rP1 homologue in binding multiple insulin-like ligands in a decapod crustacean. This constitutes further evidence for the conserved nature of the insulin-signalling system in decapod species. By employing molecular modelling approaches we have assessed the structural, but more importantly, the physicochemical nature of the IGFBP_N’. In doing so, we suggest that these physicochemical characteristics are at the core of the IGFBP_N’ divergences across species. The inverse electrostatic complementarity that we illustrate to exist between the decapod and vertebrate IGFBP_N’-ligand complexes is evidence of such. These dramatic divergences likely justify the specificity of ligand binding between Phyla.

## 4. Materials and Methods

### 4.1. Sequences

In addition to the previously described sequences for Sv-IAG (KF220491.1), Sv-ILP1 (KP006643), and Sv-IGFBP (KU195720), this work is the first to describe a second insulin-like peptide in *S.-verreauxi*, Sv-ILP2 (KP006646), as well two homologues in *C. quadricarinatus*, so named Cq-ILP1 (KP006644) and Cq-ILP2 (KP006645). Sequences were mined from transcriptomic data using a Java script for the conserved cysteine residue motif (using CLC (v7.5.1)). All sequences have been submitted to NCBI Genbank (Accession Numbers given in brackets). Phylogenetic analyses were conducted with mature ILP sequences (removal of signal and C-peptide) in Mega (7.0.21), aligned by Muscle and trees constructed using the neighbour-joining method with 1000 bootstrap replicates. This work also uses the *C. quadricarinatus* IGFBP, Cq-IGFBP (KC952011.1), and Cq-IAG (ABH07705.1). Sequence alignment and physicochemical analyses were conducted using Clustal2.1 (http://www.ebi.ac.uk/Tools/msa/clustalo/).

### 4.2. Protein Structure Modelling

Sequences for Sv-IGFBP, Cq-IGFBP, Sv-IAG, Sv-ILP1, and Sv-ILP2 were submitted to LOMETS http://zhanglab.ccmb.med.umich.edu/LOMETS [60] to select the closest resolved structures available from the Protein Data Bank (PDB) to serve as structural templates. Models were based on the following templates: Sv- and Cq-IGFBP insulin binding and kazal domains on IGFBP-rP5, also known as HTRA1 (PDB: 3TJQ_A), and the immunoglobulin domain on a myosin light chain kinase (PDB: 2CQV_A). Sv-IAG, Sv-ILP1, and Sv-ILP2 were constructed based on insulin (PDB: 2KQP_A). In addition, each sequence was analysed using Network Protein Sequence Analysis, Consensus Secondary Structure Prediction meta-server (https://npsa-prabi.ibcp.fr/cgi-bin/npsa_automat.pl?page=/NPSA/npsa_seccons.html), to detect any structural variances from the chosen template, which were then specifically applied to each sequence (refer to Figure S1 for full details on modelling procedure).

The sequence alignments were imported into Discovery Studio 4.0 (Biovia; Accelrys Inc., San Diego, CA, USA) for model construction. Each protein model was generated using the “Build Homology Model” by MODELER [61], implementing the disulfide bond criteria and any secondary structure restraints (refer to Figure S1). In the case of Sv-IGFBP, an additional *cis*-peptide bond was defined in the C’ immunoglobulin domain. In each case, the optimal model was selected *via* its lowest energy and associated DOPE score [62] (Sv-IGFBP, −14117.9; Cq-IGFBP, −8248.2; IAG, −7841.97, ILP1, −10224.5, ILP2, −6622.85). For the ILPs the C-peptide was kept intact for modelling (as it is likely to be involved with orientation and folding) and later removed. Due to the flexible structure of the IGFBP C’ immunoglobulin domain, truncated models (IGFBP_N’) consisting of the insulin-binding and kazal domains were generated for Sv (truncated at R_153_) and Cq (truncated at R_155_) and used for subsequent analyses.

### 4.3. Molecular Docking and Binding Studies

For interaction studies, Sv-IGFBP_N’ and each ligand were imported to the Matchmaker module in UCSF Chimera (http://www.rbvi.ucsf.edu/chimera) and aligned to the resolved, bound structure of IGFBP4_N’ and IGFI (PDB: 1WQJ). The resulting bound models were saved relative to the template and reimported to Discovery Studio. Each complex was then individually refined by energy minimisation (using the CHARMm force field) to reduce steric clashes. For interaction assessment, refined complexes were then submitted to PDBsum Generate, (http://www.ebi.ac.uk/thornton-srv/databases/pdbsum/Generate.html), PRODIGY [43,44] (http://milou.science.uu.nl/services/PRODIGY/), CCharPPI [63] (https://life.bsc.es/pid/ccharppi), and then reanalysed manually in Chimera to validate interacting residues. To assess the reliability of our modelled complexes, structures were also submitted to HADDOCK2.2: Easy interface (http://milou.science.uu.nl/services/HADDOCK2.2) [64,65] to generate comparative docked complexes.

### 4.4. Alanine Scanning and Hotspot Residues

We employed computational alanine scanning to determine the hotspot residues of Sv-IGFBP_N’. This was done by replacing each residue in turn with alanine (the smallest most inert amino acid) and assessing for a significant decrease in the binding free-energy [46]. We submitted each refined bound complex to five software platforms to gain a meta-style output: Cpclab (http://cpclab.uni-duesseldorf.de/dsppi/) [66]; Robetta (http://www.robetta.org/alascansubmit.jsp) [67]; KFC (http://kfc.mitchell-lab.org/) [68]; ANCHOR (http://structure.pitt.edu/anchor/upload/); and HotRegion (http://prism.ccbb.ku.edu.tr/hotregion/) [69]. In addition, the electrostatic interaction of each Sv complex, Cq-IGFBP_N’ and a range of vertebrate structures was determined by performing electrostatic potential surface calculations using PDB2PQR [70] and APBS [71] programmes within UCSF Chimera (with protonation states at physiological pH and 298 K and parse charges). The surface potential representation is shown in each figure, with charge levels ranging from −kT/e (red) to +kT/e (blue), as indicated by the scale bar.

## Supplementary Materials

Supplementary materials can be found at www.mdpi.com/1422-0067/18/9/1832/s1.

## Abbreviations

AG	Androgenic gland
C’	C terminal
Cq	*Cherax quadricarinatus* (red-claw crayfish)
Dilp7	*Drosophila* ILP7
Dilp8	*Drosophila* ILP8
IAG	Insulin-like androgenic gland peptide
IGF	Insulin-like growth factor
IGFBP	Insulin-like growth factor binding protein
IGFBP-rP1	Insulin-like growth factor binding- related protein
ILP	Insulin-like peptide
N’	N terminal
RPKM	Reads per kilobase per million reads
Sv	*Sagmariasus verreauxi* (Eastern spiny lobster)
TKIR	Tyrosine kinase insulin receptor
