# Supplementary material for: Understanding Insulin Endocrinology in Decapod Crustacea: Molecular Modelling Characterization of an Insulin-Binding Protein and Insulin-Like Peptides in the Eastern Spiny Lobster, Sagmariasus verreauxi
Source: Int J Mol Sci. 2017 Aug 23;18(9):1832. doi: 10.3390/ijms18091832 (PMC5618481; doi:10.3390/ijms18091832)
Supplement: Supplementary file 1 [file ijms-18-01832-s001.pdf]

Supplementary Materials: Understanding Insulin Endocrinology in Decapod *Crustacea*: Molecular Modelling Characterization of an Insulin-Binding Protein and Insulin-Like Peptides in the Eastern Spiny Lobster, *Sagmariasus verreauxi*

Figure S1: Modelling criteria for (a) Sv-IGFBP; (b) Cq-IGFBP_N′; (c) Sv-IAG; (d) Sv-ILP1; (e) Sv-ILP2. In each case the trimmed modelled sequence is given (subscript numbering in brackets indicating the native amino acid position) with the following associated details listed: (i) model templates; (ii) input sequence alignments; (iii) secondary structure predictions and resulting additional restraints (implemented due to deviation from the chosen template), also shown in sequence in red bold; and (iv) defined bonds and any additional refinement. Key as shown, colouration as previously described.

Alpha helix	(**Hh**)
3_{10} helix	(**Gg**)
Pi helix	(**Ii**)
Beta bridge	(**Bb**)
Extended strand	(**Ee**)
Beta turn	(**Tt**)
Bend region	(**Ss**)
Random coil	(**Cc**)
Removed in refinement	(~~strike~~)

(a) Sv-IGFBP

$_{(21)1}$~~*QDVVT*~~E**C**GE**C**DRSN**C**PEVKT**C**LGGKVQDA**C**G**CC**EV**C**ALGLGQR**C**DSQDSGDSTDYGS**C**GEYLV**C**RTRTDTG GTDEAT**C**V**C**ENPDPV**C**GSDGITYSTL**C**HLLQETTEKPDVFVAVRGP**C**KGVPVIKSKPEDKIRPLGSILVLD**C**EAA GYPVPEITWELNRPDGSTMKLPGDDSSFAVQVRGGPEDHMVTGWVQIMRITKKSLGIYT**C**VATNTEGETRVSATV ALKEHGEKEDSMNKL$_{236(256)}$

(i) Model template selections

Insulin-binding + kazal domains: HTRA1 (3TJQ_A)

Immunoglobulin domain: Myosin light chain kinase (2CQV_A)

(ii) Input sequence alignments

```
Sv-IGFBP      1  QDVVTECG-ECDRSNCPEV-KTCLGGKVQDACGCCEVCALGLGQRCDSQDSGDSTDYGSCGEYLVCRTRT  68
3TJQ_A       13 FQGSAGCPDRCEPARCPPQPEHCEGGRARDACGCCEVCGAPEGAACGLQ-------EGPCGEGLQCVVPF  75
3TJQ SS Pred    ccCCCCCCCCCChhhCCCCCCCCCCcccCCCCCcCccccccccCCCCCCC-------CCCCCCCCceeCCC

Sv-IGFBP     69 D-------TGGTDEATCVCENPDPVCGSDGITYSTLCHLLQET-TE----KPDVFVAVRGPCKGV       121
3TJQ_A       76 GVPASATVRRRAQAGLCVCASSEPVCGSDANTYANLCQLRAASRRSERLHRPPVIVLQRGACGNS       140
3TJQ SS Pred    CCCcchhhhhhccCCeEeecccCCeeecCCcEECCHHHHHHHHHhccccCCCCeEEeeecCCCCC

Sv-IGFBP    122 PVIKSKPEDKIRPLGSILVLDCEAAGYPVPEITWELNRPDGSTMKLPGDDSSFAVQVRGGPEDHMVTGWV  191
2CQV_A        8 PQIIQFPEDQKVRAGESVELFGKVTGTQPITCTWMKFRKQIQ------ESEHMKVENSEN------GSKL  65
2CQV SS Pred    CeEeeCCCceEEeCCCcEEEEEEEEecCCCEEEEEeCCeecc------ccccEEEEEcCC------ceEE

Sv-IGFBP    192 QIMRITKKSLGIYTCVATNTEGETRVSATVALKEHGEKEDSMNKL                           256
2CQV_A       66 TILAARQEHCGCYTLLVENKLGSRQAQVNLTVVDKPDPPAGTPSG                           110
2CQV SS Pred    EEecccchhhCEEEEEEEEecCccccEEEEEEEEecCCCCCCCCCCC
```

(iii) Additional secondary structure restraints

Secondary structure predictions fit model templates, no restraints applied.

```
                    10        20        30        40        50        60        70
                     |         |         |         |         |         |         |
Sv-IGFBP   QDVVTECGECDRSNCPEVKTCLGGKVQDACGCCEVCALGLGQRCDSQDSGDSTDYGSCGEYLVCRTRTDT
DPM        cceeeetccttttctcceeeetcccecthtcceheeheccctcctcttttttttcccttttcceeeeeeecccc
DSC        cccccccccccccccccccccccceeeccccccccccccccccccccccccccccccccceeeeeeeccc
GOR4       cccccccccccccccccceeeeeccccccccccceeeccccccccccccccccccccceecccceeeeeccccc
HNNC       cceeeeccccccccccccccccccccccccccceeeeccccccccccccccccccccccceeeeeeeecccc
PHD        ccccccccccccccccccccccccccccccccccccceccccccccccccccccccccccccceeeccccc
Predator   ccceeeccccccccccccccccccccccccccceeeeeccccccccccccccccccccccceeeeeeecccc
SIMPA96    cceecccccccccccccccccccccccccccccccccccccccccccccccccccccccceeeeeccccc
SOPM       heeeeeccccccccccchhhhhtcccchtttcceeeeeeccccccccccccccccttttteeeeeeccccc
Sec.Cons.  cc?e??ccccccccccccccccccccccccccceeeccccccccccccccccccccccccccceeeee?cccc

                    80        90       100       110       120       130       140
                     |         |         |         |         |         |         |
Sv-IGFBP   GGTDEATCVCENPDPVCGSDGITYSTLCHLLQETTEKPDVFVAVRGPCKGVPVIKSKPEDKIRPLGSILV
DPM        ctccchheeeecttccccctttcceeeeeeeeeehhhccctcceeeeeeetctccceceeeccttcccecccceeee
DSC        cccccceeeeecccccccccccceeeeeeeeehhhhcccccceeecccccccccceeecccccceeeccceee
GOR4       ccccceeeeeccccccccccccceecchhhhhccccccccceeeeeecccccccceecccccccccccccceee
HNNC       cccccceeecccccccccccccceeeehhhhhccccccccceeeeecccccccceeeeccccccccccccheee
PHD        ccccceeeeecccccceeeccccceeecchhhehhhhccccceeeccccccccccceeccccccccccccceee
Predator   ccccceeeeeccccccccccccccceeehhhhhhhcccccceeeeeecccccceeeeccccccccccccceee
SIMPA96    ccccceeeeecccccccccccccchhhhhhhhhhcccccceeeeeccccccceeeccccccccccccceeee
SOPM       ccccchheeeeccccccccccttcchhhhhhhhhhhttcccceeeeeccccccceeeccccccccccccceeee
Sec.Cons.  ccccceeeeecccccccccccccceeeehhhhhhhcccccceeeeeccccccc?ee?ccccccccccccceee

                   150       160       170       180       190       200       210
                     |         |         |         |         |         |         |
Sv-IGFBP   LDCEAAGYPVPEITWELNRPDGSTMKLPGDDSSFAVQVRGGPEDHMVTGWVQIMRITKKSLGIYTCVATN
DPM        ehhhhcccccccceeehhctttcccccctttttcceeeeeetctcccceeeeeeeeeeeeeccceceeeeeeeet
DSC        eeeeecccccceeeeeeeeccccccccccccccceeeeeeccccccceeeeeeeeeeecccccccceeeeeeec
GOR4       eeeccccccccccceeeeeccccccceeeccccccceeeeecccccceeeeeeeeeeeeeeeeeeeeccceeeeeeeec
HNNC       eeccccccccccceeeeccccccccccccccccccceeeeeccccccchhhhhhhhhhhhcccceeeeeeeecc
PHD        eeeeeecccccceeeeeeeeccccccccccccccceeeeeeccccccceeeeeeeeeeeccccccceeeeeeeec
Predator   eeccccccccccccceeecccccccceeeccccccceeeeeecccccccccceeeeeeccccccceeeeeeecc
SIMPA96    eeccccccccccceeeeeccccccceeeeccccccceeeeeccccccceehhhhhhhhhhhccccceeeeeeecc
SOPM       eectttttcccccceeeeeccttcceeeccccccceeeeeetcccccccceeechhhhhhhcccccceeeeeeecc

Sec.Cons.  eecccccccccceeeeecccccccc??cccccccceeeeeccccccceeeeeeeeee?ccccccceeeeee?c

                   220       230
                     |         |
Sv-IGFBP   TEGETRVSATVALKEHGEKEDSMNKL
DPM        ccccceeeeeeehhhctccccccthccc
DSC        cccceeeeeeeeeeeecccccccccccc
GOR4       ccccchhhhhhhhhhhccccccccccee
HNNC       cccchhehhhhhhhhhcccccccccccc
PHD        cccceeeeeeeeeeeecccccccccccc
Predator   ccccchhhhhhhhhhhcchhhhhhcccc
SIMPA96    ccccchhhhhhhhhhhccccchhhhhhc
SOPM       cttccchhheeehhhttcchhhhhhhh
Sec.Cons.  ccccch?hh??hhhhccccccccccccc
```

(iv) Defined bonds and refinement

Insulin-binding domain: C_{7}-C_{30}; C_{10}-C_{32}; C_{15}-C_{33}; C_{21}-C_{36}; C_{58}-C_{78}; C_{44}-C_{64}; C_{98}-C_{80}

Kazal and immunoglobulin domain: C_{118}-C_{87}

Immunoglobulin domain: C_{143}-C_{206}; Cis-peptide bond P_{149}

${}_{1}$QDVVT$_{5}$ removed during refinement due to steric clashes.

(b) Cq-IGFBP_N′

(25) 1 TCGECDRSKCAVTASCPGGLVLDTCGCCEVCARGLGQLCENTTTEDTPTTTHYGVCGQYLVCRTRTDTGGT GEATCECEDSGAVCGSDGVTYTTLCHLLMETAERPDLSVAVRGPCKTAPVIKSKPEDKIR 131 (155)

(i) Model template selections

Insulin-binding + kazal domains: HTRA1 (3TJQ_A)

(ii) Input sequence alignments

```
Cq-IGFBP_N’    1 TCG-ECDRSKCAVT-ASCPGGLVLDTCGCCEVCARGLGQLCENTTTEDTPTTTHYGVCGQYLVCRTRTD-   67
3TJQ_A        18 GCPDRCEPARCPPQPEHCEGGRARDACGCCEVCGAPEGAACGLQ----------EGPCGEGLQCVVPFGV   77
3TJQ SS Pred     CCCCCCChhhCCCCCCCCCCcccCCCCCcCcccccccCCCCCCC----------CCCCCCCCceeCCCCC

Cq-IGFBP      68 ------TGGTGEATCECEDSGAVCGSDGVTYTTLCHLLMETA-E----RPDLSVAVRGPCKTA          119
3TJQ_A        78 PASATVRRRAQAGLCVCASSEPVCGSDANTYANLCQLRAASRRSERLHRPPVIVLQRGACGNS          140
3TJQ SS Pred     CcchhhhhhccCCeEeecccCCeeecCCcEECCHHHHHHHHHhccccCCCCeEEeeecCCCCC
```

(iii) Additional secondary structure restraints

Secondary structure predictions fit model template, no restraints applied.

```
                        10        20        30        40        50        60        70
                         |         |         |         |         |         |         |
Cq-IGFBP_N'TCGECDRSKCAVTASCPGGLVLDTCGCCEVCARGLGQLCENTTTEDTPTTTHYGVCGQYLVCRTRTDTGG
DPM        ctccttttceheeccttcceeececceeheehccccchtcccccccccccceteeteeeeeeeecccctt
DSC        ccccccccccccccccccccccccccccccccccccccccccccccccccccccccccceeeeccccccc
GOR1       eettttttheeeeeeetteeeeetttteeeehtttceeeeeccctttcttеееееееееееееееtttct
GOR3       cccccccccceeccccccccеесссссссссссссceeeecccccccccеееесеесеееееееессссс
HNNC       ccccccccceeeeecccccеееесссссеесhcccccccccccccccccccеееееceeeeeeecccccc
MLRC       ccccccccccccccccccceeeccccccccccccccccccccccccccccccccccccceeeeeccccccc
PHD        ccccccccccccccccccceeccccccceeeccccccecccccccccccccccccccccеееесссссссс
Predator   ccccccccceeeeccccccеееcccccеееcccccccccccccccccccеееcccccеееееееcccccc
SOPM       ccccccccceeeeeccttceeeecccccchhhhhhhhhhcccccccccccccеееееееееееесссссссс
Sec.Cons.  ccccccccceeeeccccccеееccccccеесссссссссссссссссссссееceeceeeeeeecccccc

                        80        90       100       110       120       130
                         |         |         |         |         |         |
Cq-IGFBP_N'TGEATCECEDSGAVCGSDGVTYTTLCHLLMETAERPDLSVAVRGPCKTAPVIKSKPEDKIR
DPM        ccchchhtttcccetttcceeeeeeeeeehhhhhcccceeeeetcccccceeccttccccc
DSC        cccceeeeeccccccccccccccceeeehhhhhhccceeeeccccccccceeecccccccc
GOR1       ccceeehhhhhhheeeetteeeeeeehhhhhhhhhhhheeeeeetteeeeeeeehhhhhhh
GOR3       ccceeeccccccceeecccccеееhhhhhhcchcccccceeeccccccccccccccccchhh
HNNC       cccccccccccccccccccceeeeeehhehccccccceeeeeccccccccееccccccccc
MLRC       ccceeeeeccccceccccccchhhhhhhhhhhhcccceeeeecccccccceeecccccccc
PHD        ccceeeeecccceeecccccceehhhheeeeecccceeeeeeccccccccеееcccccccc
Predator   cccceeeccccccccccccccchhhhhhhhhhhccccceeeeeсссссссссссссссссс
SOPM       cccceehhccttеееccttceeehhhhhhhhhttcccceeeecccccccceeeccccccce
Sec.Cons.  ccc?eee?ccccce?ccccccee?hhhhhhhhhcccc?eeeeccccccccеееcccccccc
```

(iv) Defined bonds and refinement

Insulin-binding domain: C_2-C_{25}; C_5-C_{27}; C_{10}-C_{28}; C_{16}-C_{31}; C_{39}-C_{62}; C_{56}-C_{76}; C_{78}-C_{96}

Kazal domain: C_{85}-C_{116}

(c) Sv-IAG

(23) 1 YNVSGLSEDFE**C**GDFENVLGRI**C**AETQSNIVRDTRSVSTVAVADSTHGGTDPSRRPYHHPRAIQVVLRHAA
NPPATQGAGAEEGVRVTSEAAFSLVKSRSIRDTRETNLQDE**CC**PFPLVH**C**DKEEILHY**C**FLTEG 135 (157)

(i) Model template selections

Insulin: (2KQP_A)

ii) Input sequence alignments

```
Sv-IAG         7 SEDFECG-DFENVLGRICAETQSNIVRDTRSVSTVAVADSTHGGTDPSRRPYHHPRAIQVVLRHAANPPA  69
2KQP_A         9 VNQHLCGSDLVEALYLVCGERGFFYTKP--------------------TRREAEDLQVGQVE---------  50
2KQP SS Pred     CCccccchhHHhHHhHHhhcCcCeecCCC--------------------CccccchhhhhccccC---------

Sv-IAG        70 TQGAGAEEGVRVTSEAAFSLVKSRSIRDTRETNLQDECCPFPLVHCDKEEILHYCF                125
2KQP_A           -LGGGPGAGSL----QPLAL----EGSLQKRGIVEQCC---TSICSLYQLENYCN                 86
2KQP SS Pred     -cCCCCCCccc----hhHHh----hccccCCchHHhc---CCCCCHHHHHHhcc
```

(iii) Additional secondary structure restraints

Additional restraints applied based on secondary structure predictions

C-peptide (later removed): A_{62}-R_{68}; $G_{80-}A_{92}$: helix restraint

```
                          10        20        30        40        50        60        70
                           |         |         |         |         |         |         |
Sv-IAG        YNVSGLSEDFECGDFENVLGRICAETQSNIVRDTRSVSTVAVADSTHGGTDPSRRPYHHPRAIQVVLRHA
DPM           cteccctcchhtcchcceechehhhhhcceeeceeeeeeeeeehctctctcttt cccccccchheeeeehhh
DSC           ccccccccccccccccccccccccccccccccccccceeeeeeecccccccccccccccccccchheehhc
GOR1          eeecccchhhhhhhhhhhhhhheeehhtteeeeeeeeeeeeeeeettttcccteettcccthheeeeeec
GOR3          eecccccccccccccchcceeeeehccccceeeeeeeceeeeeeeeecccccccccccccccchhheehhhc
HNNC          ccccccccccccccchhhhheeeecccccceeeccceeeeeeecccccccccccccccccchhhhhhhhhc
MLRC          cccccееcccccccchhhhhhhhhhcccccccccccccceeeeecccccccccccccccccccchheehhhc
PHD           cceeeeeecccccccchhhhhhhhhcccccccccccccccceeecccccccccccccccccccceeeeeecc
Predator      cccccccccccccccchhhhhhhcccccccccccccceeeeeeccccccccccccccccchhhhhhhhhc
SOPM          echhccchhccttchhhhhhhhhhhcchheeccttceeeeeeecccccccccccccccccchhheeeeecc
Sec.Cons.     ccccccccccccccchhhhhh?hhccccceeccccccceeeeeeccccccccccccccccc?hhheeehhc
```

```
                          80        90       100       110       120       130
                           |         |         |         |         |         |
Sv-IAG        ANPPATQGAGAEEGVRVTSEAAFSLVKSRSIRDTRETNLQDECCPFPLVHCDKEEILHYCFLTEG
DPM           htccccctcchhhceeeehhhhhhheetheehcchhcchhthccccceehhhhhhhhheeeeecc
DSC           cccccccccccccceeeehhhhhhhhhhhhhhhccccccccccccccccccchhhhhhheecccc
GOR1          ccccceeecchhhhhhhhhhhhhhhhhceeeeecttttttttttetehhhhhhhhhhhhhhhhhht
GOR3          ccccchhcccceeeeeeehhhhhhheehcececcccccccccccccccccccchhhhehhecccc
HNNC          cccccccccccchhhhhhhhhhhhhhhhcccccccccccccccccccccccchhheeeeeeeccc
MLRC          ccccccccccchchchhhhhhhhhhhhhcccccccccccccccccccccccchhhhhhhhhhccc
PHD           ccccccccccceceeeechhhhhhhhhhccccchhhccccccccccccccccchhhhhhheceecc
Predator      cccccccccccccccchhhhhhhhhhhcccccchhhhhhccccccccccccccccceeeeeeccc
SOPM          ccccccccccchttceeehhhhhhhhhcccccccccccccccccccceeecctthhhhhhhhhhct
Sec.Cons.     ccccccccccchc?eee?hhhhhhhhh?ccccccccccccccccccccccccchhhhhhheeeccc
```

(iv) Defined bonds and refinement

C_{12}-C_{114}; C_{23}-C_{130}; C_{113}-C_{121}

(d) Sv-ILP1

24 LEPDLISQIESRTEKEWQELWTEERLTL**C**RSRLRHNLDAI**C**GKDVYRRSSMLPPRTRHRRWSRAKRNTDIFLE
VHDTDTARGDSRKKEKRMKTMSVDLPTTRIEISPSVPDTGQHSTHTRSPFLSVHQANLFVTTWVGGHHRHRRQSP
SITSE**CC**TTVG**C**TWEEYAEY**C**PTSSRLRPGVTLI 205

(i) Model template selections

Insulin: (2KQP_A)

(ii) Input sequence alignments

```
Sv-ILP1        24 ERLTLCRSRLRHNLDAICGKDVYRRSSMLPPRTRHRRWSRAKRNTDIFLEVHDTDTARGDSRKKEKRMKT  93
2KQP_A          2 VNQHLCGSDLVEALYLVCGERGFFYTKP----TR-------REAEDLQVGQVEL----------------  44
2KQP SS Pred      CCccccchHHHHHHHHHhcCcCCCCCCC----Cc-------ccchhhhcccccc----------------

Sv-ILP1        94 MSVDLPTTRIEISPSVPDTGQHSTHTRSPFLSVHQANLFVTTWVGGHHRHRRQSPSITSECCTTVGCTWE  163
2KQP_A         45 ----------------------GGGPGAGSLQPL------------ALEGSLQKRGIVEQCCT-SICSLY  79
2KQP SS Pred      ----------------------CCCCCCccchhH------------HhhcccccCCchHHhcC-CCCCHH

Sv-ILP1       164 EYAEYCP                                                                 170
2KQP_A         80 QLENYCN                                                                 86
2KQP SS Pred      HHHHhcC
```

(iii) Additional secondary structure restraints

Additional restraints applied based on secondary structure predictions

B-chain: P_3-C_{41}: helix restraint

A-chain: T_{162}-Y_{168}: helix restraint

C-peptide (later removed): R_{60}-R_{66}; S_{84}-M_{94}: helix restraint

NB: note the highly disordered nature of the A-chain, predicted as random coil

```
                   10        20        30        40        50        60        70
                    |         |         |         |         |         |         |
Sv-ILP1    LEPDLISQIESRTEKEWQELWTEERLTLCRSRLRHNLDAICGKDVYRRSSMLPPRTRHRRWSRAKRNTDI
DPM        ctccheeeehhhhhhhhhhhhhhhhheeehhhhhhchhhecticceeeecccccccchhhhhhhhhhtcce
DSC        ccccceeeeecccccchhhhhhccccccccchhhhheeeccccccccccccccccccchhhhhcccccce
GOR1       hhhhhhhhhhccchhhhhhhhhhhhhhhhhheettteeeeeeeeeeeeetccctteeettttcttttccee
GOR3       cccchhhhhhhhhhhhhhhhhhhhhhhhhhhhcccccchhccccceceecccccccceeeeeehhccchhh
HNNC       cccchhhecccccchhhhhhhhhhhhhhhhhhhhccccchecccchhhcccccccccchhhhhhhhcccce
MLRC       ccchhhhhhhhhhhhhhhhhhhhhhhhhhhhhhhcccchcccccceeccccccccccccccchhhcccee
PHD        cccceecccccchhhhhhhhhhcccchhhhhhhhhhhhhhhhccceeeccccccccccccccccccccce
Predator   cccchhhhhhhhhhhhhhhhhhhhhhhhhhhchhhhhccccccccccccccccccccchhhhhhhhhhhe
SOPM       ccthhhhhhhhhhhhhhhhhhhhhhhhhhhhhhhhhhhhhttcceeeccccccccccchhhhcccccee
Sec.Cons.  cccchhhhhhhhhhhhhhhhhhhhhhhhhhhhhhh?hch?ccccceeccccccccccchhhhhh?cccce

                   80        90       100       110       120       130       140
                    |         |         |         |         |         |         |
Sv-ILP1    FLEVHDTDTARGDSRKKEKRMKTMSVDLPTTRIEISPSVPDTGQHSTHTRSPFLSVHQANLFVTTWVGGH
DPM        eehehcccchttttccchhhhheeeecccceeeheecccccctcccccccccceeeehhhheeeeeeeccc
DSC        eeeecccccccccccchhhhheeeeeeccccceeeeeccccccccccccccccccceeecccceeeeeeeccc
GOR1       eeehhhhhhhhhhhhhhhhhhheeeeecceeeeeeetccctcteeeeetcctteeeehhheeeeeeectcc
GOR3       ehheccccchhccchhhhhhhhhheccccccceeeecccccccceeceeceeceeehhhhhhheeeeeeecc
HNNC       eeeeccccccccccchhhhccceeeccccccceeeeccccccccccccccccccceeecccceeeeeeeccc
MLRC       eeeeeccccccccchhhhhhhhhhhcccccccceccccccccccccccccceeeecccceeeeeccccc
PHD        eeccccccccccccccccccccccccccccccccccccccccccccccccceeeecchhheeeeeccccc
Predator   eeeecccccccccchhhhhhhhhhcccccccceeeecccccccccceeecccccccccccccceeeecccc
SOPM       eeeeeccccttcchhhhhhhheeeeccccccceeccccccccccccccccccceeeeccttteeeeeeetcc
Sec.Cons.  eeeecccccccccchhhhhhhheeeccccccceeeecccccccccccccccccceeeec??ceeeeeeeccc
```

```
                  150       160       170       180
                    |         |         |         |
Sv-ILP1    HRHRRQSPSITSECCTTVGCTWEEYAEYCPTSSRLRPGVTLI
DPM        thhhhctcceeeheeeeeeeehhhhhhcccctccctcceecc
DSC        ccccccccceeeeeeccccccccccccccccccccccccceec
GOR1       ccttttcttteeeetttetcthhhhttttttteeeeeteeee
GOR3       ceeecccccceeeeeeeccccccccccccccccccccceeee
HNNC       ccccccccccccccccccccchcccccccccccccccccccc
MLRC       ccccccccccccccccccccchhhhhhcccccccccccceec
PHD        ccccccccccchhhccccccchhhhhhcccccccccccceec
Predator   cccccccccccccccccccccccccccccccccccccccccc
SOPM       ccccccccccccccccettcchhhhhhccttccccctttceee
```

(iv) Defined bonds and refinement

C_{29}-C_{155}; C_{41}-C_{169}; C_{154}-C_{160}

(e) Sv-ILP2

$_{21}$RPYEETRSYKICTSRDVKVMANYVCNLHRRRRSVLSLDDARDNYGVPGLLLENRSRRLALPQHWRPEDDTDTG
NVSRRDPSFLQFTRIRRQVLLGEIRKQCCVHGCTPRDFYGACQ$_{136}$

(i) Model template selections

Insulin: (2KQP_A)

(ii) Input sequence alignments

```
Sv-ILP2        8 SYKICTSRDVKVMANYVCNLHRRRRSVLSLDDARDNYGVPGLLLENRSRRLALPQHWRPEDDTDTGNVSR   77
2KQP_A         3 NQHLCGSDLVEALY-LVC------------------GERGFFYTKPTRR--------EAEDLQVGQVEL   44
2KQP SS Pred     CccccchHHHHHHHHHc------------------CcCeecCCCCcc--------cchhhhcccccC

Sv-ILP2       78 R---DPSFLQFTRIRRQVLLGEIRKQCCVHGCTPRDFYGAC                                115
2KQP_A        45 GGGPGAGSLQPLALEGSLQKRGIVEQCCTSICSLYQLENYC                                85
2KQP SS Pred     CCCCCccccCCcHhhcccccCCchHHhcC-CCCCHHHHHHhc
```

(iii) Additional secondary structure restraints

Additional restraints applied based on secondary structure predictions

B-chain: V_{17}-R_{29}: helix restraint

A-chain: Q_{100}-V_{103}; P_{108}-F_{111}: helix restraint

C-peptide (later removed): N_{53}-A_{59}: helix restraint

NB: note the very short helix of the alpha chain won't make a full helix, rather an alpha turn

```
                      10        20        30        40        50        60        70
                       |         |         |         |         |         |         |
Sv-ILP2     RPYEETRSYKICTSRDVKVMANYVCNLHRRRRSVLSLDDARDNYGVPGLLLENRSRRLALPQHWRPEDDT
DPM         tcchhchceeeeeceeehehhheeechhhhhheeehhhthtttcccccchhhthhhhhhhcchccctccc
DSC         cccccccccceeeccccceeeeehhcchhcccceeeccccccccccccceeehhhhhhhccccccccccc
GOR1        cchhhhttteeeeeeheeeeehhheeeeeeeeeeeeeeeeetttteeeeeeehhhhhheeecttccctttt
GOR3        cccceceeeeeeecccccceeeehhheccccccceeeeeeccccccccccceeehhhhhhhhhcccccccccc
HNNC        cccccccceeeeeecccchhhhhhhhhhhhccccceeeecccccccccccceeecccccceccccccccccc
MLRC        cccccccceeeecccchhhhhhhhhhhhhhhhccceeeeeecccccccccccceeeccccccccccccccccccc
PHD         cccccccceeeeeeeeechhhhhhhhhhhccccceeeeeeccccccccccceeeeecccceccccccccccccc
Predator    ccccccccccccccccceeeeccccccccccccccccccccccccccccchhhhhhhhcccccccccccc
SOPM        ccttcccceeeecchhhhhhhhhhhhhhhhtcceeeeeehhhttttccctteeecccccccccccccccccc
Sec.Cons.   ccccccc?eeeecccccceehhhhhh?hhcccceeeeeeccccccccccccceeeh?hhhhhcccccccccccc

                      80        90       100       110
                       |         |         |         |
Sv-ILP2     DTGNVSRRDPSFLQFTRIRRQVLLGEIRKQCCVHGCTPRDFYGACQ
DPM         ccctettcccchhheehehhheehchhhhheeeccccccccccchcc
DSC         cccccccccccchhhhhhhhhhhhcccccceeeecccccccccccccc
GOR1        ttteeetecttteeeeeeeeeeeeeethhhteeeetteetttttteee
GOR3        ccccceccccchheehhhhhhheeccccccceeeecccccccccccccee
HNNC        cccccccccccchhhhhhhhhhhhhhhhchceeccccccccccccccc
MLRC        cccccccccccchhhhhhhhhhhhhhhhhhhccccccccccccccccc
PHD         cccccccccccccchhhhhhhhhhhhhhhhhheeeccccccccccccc
Predator    cccccccccccccchhhhhhhhhhhhhhhhhccccccccccccccccc
SOPM        cccccccccccceeeehhhhhhhhhhhhcttceettcccchechhh
Sec.Cons.   cccccccccccchhhhhhhhhhhhhhhhhhh?eecccccccccccccccc
```

(iv) Defined bonds and refinement

C_{12}-C_{102}; C_{25}-C_{115}; C_{101}-C_{106}

Figure S2. PDB predicted interactions of A and B-chains of IAG, ILP1 and ILP2. Interaction map describing residue specific interactions between A- and B-chains of each ligand. Standard amino acid abbreviations are used, with colours indicating physicochemical properties as follows: blue- positive, red- negative, green- neutral, grey- aliphatic, mauve- aromatic, orange- proline and glycine, yellow- cysteine. Number of interacting residues given in brackets.

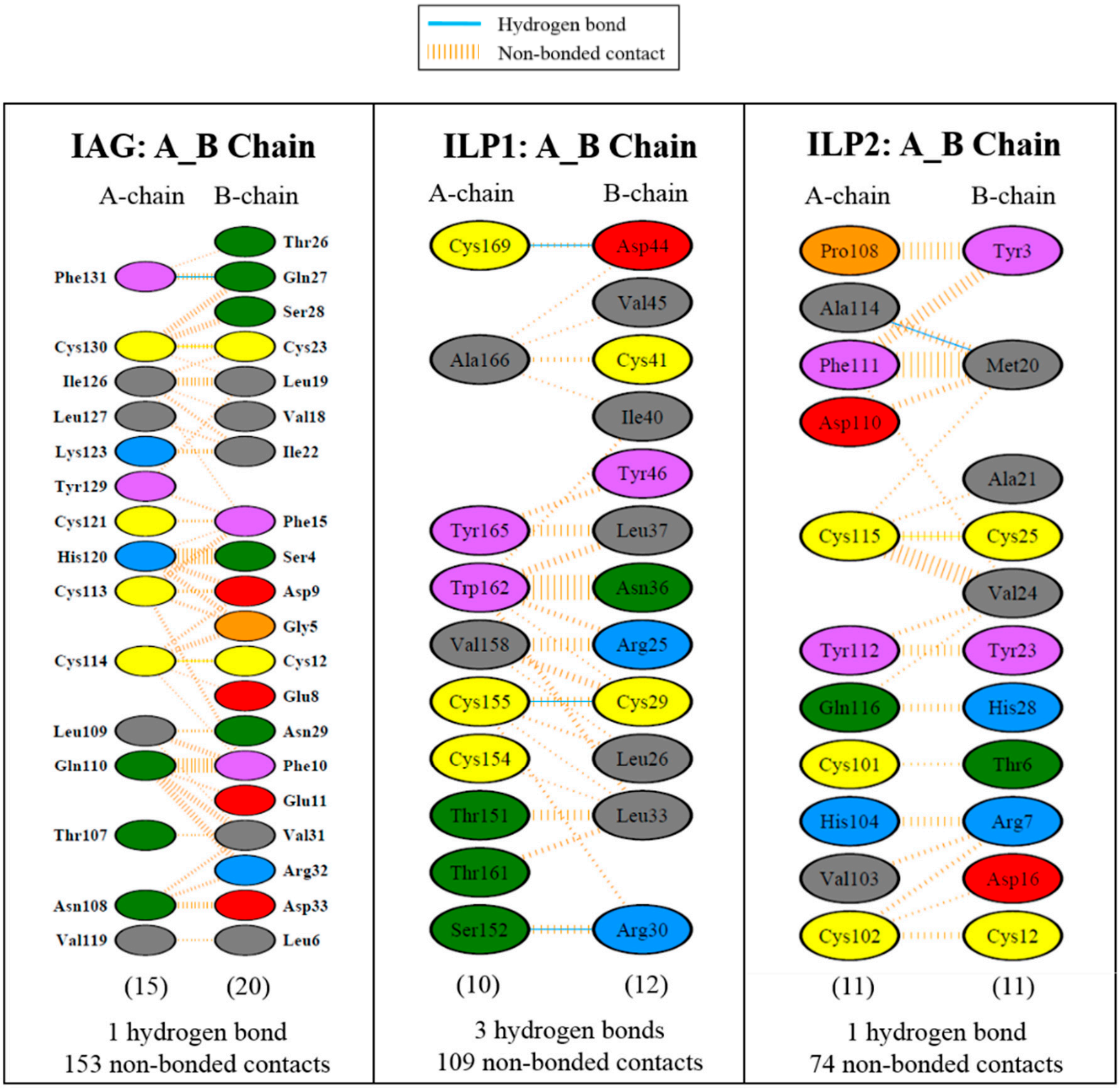

Figure S3. HADDOCK2.2 molecular docking as assessment of reliability of complex formation. Structural alignment of Sv complexes (darker shade) with the best-fit models generated by Haddock (lighter shade), shown in ribbon format. Good alignment suggests reliability of our generated complexes.

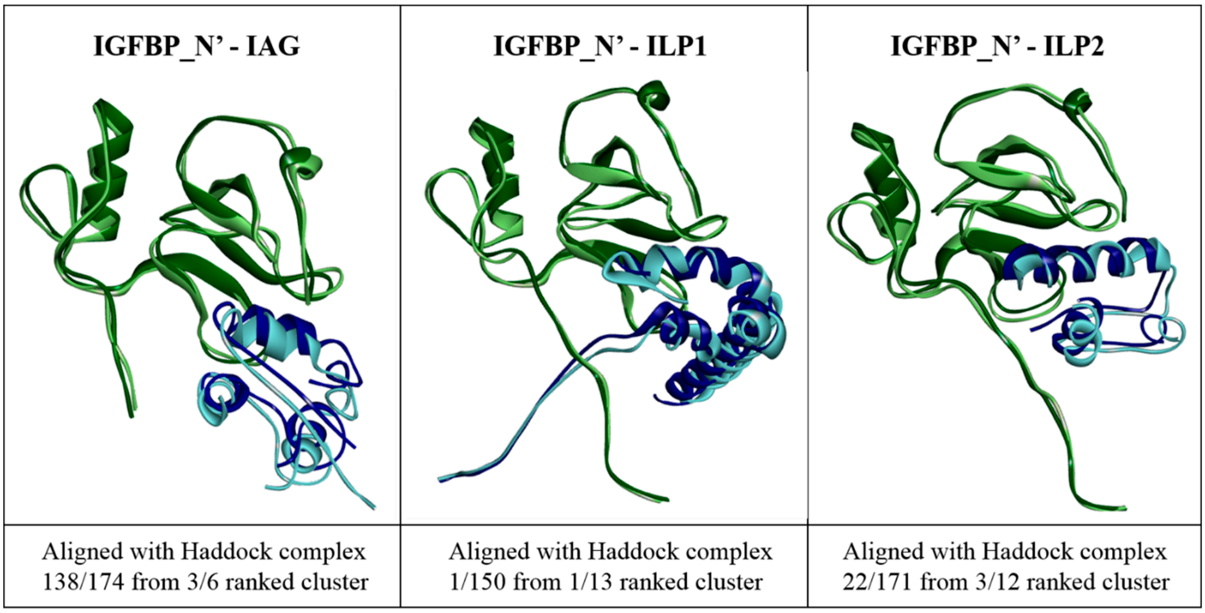

Figure S4: Electrostatic potential surface of human IGFII (PDB: 2L29). Binding orientation depicted, with the binding interface bracketed. Surface is coloured by potential on the solvent accessible surface on a scale of -kT/e (red) to +kT/e (blue), as indicated by the scale bar.

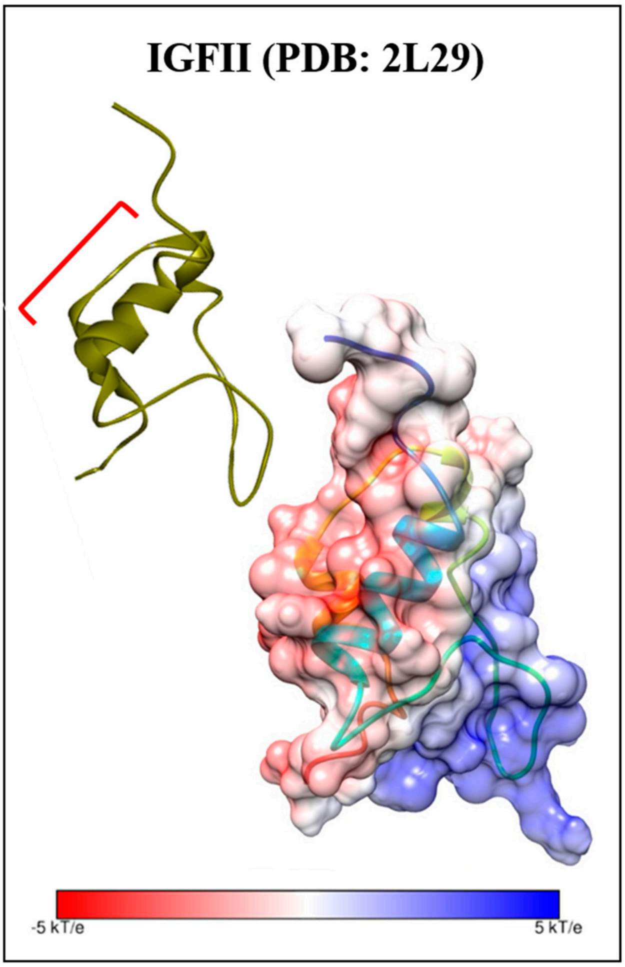

Table S1: PRODIGY predicted interactions of Sv-IGFBP_N′ and all ligands, complementary to PDBsum predictions. a) All residue contacts of IGFBP_N′ (I chain) and ligand (A, orange and B, blue chains) listed with text in the corresponding colours. Text box colouration is used to represent the physicochemical properties (red- negative, green- neutral, grey- aliphatic, orange- proline and glycine, yellow- cysteine) of the consistently interacting contacts of Sv-IGFBP_N′. The number of predicted contacts of these residues is given, with the average as indication of the weighted importance of the residue. b) Comparison of interaction contacts predicted by PRODIGY and PDBsum and the PRODIGY description of the physicochemical nature of these contacts. c) Summary of the consistently interacting contacts of the IGFBP_N′ interface, predicted by both PRODIGY and PDBsum. ‘All’ indicates a conserved prediction of an interaction with all three ligands by both PRODIGY and PDBsum, otherwise the ligand(s) with which PDBsum predicts an interaction is shown.

(a)

IAG - IGFBP_N'	n contact	ILP1 - IGFBP_N'	n contact	ILP2 - IGFBP_N'	n contact	Consistent	Average n contacts
ALA 49 I ALA 24 B		ALA 49 I ASP 38 B		ALA 49 I ASN 22 B			
ALA 49 I ASN 17 B		ALA 49 I GLY 42 B		ALA 49 I ASN 26 B			
ALA 49 I GLU 25 B	3	ALA 49 I HIS 35 B	3	ALA 49 I CYS 25 B			
ASP 65 I ARG 21 B	1	ASP 68 I ARG 32 B	1	ALA 49 I LYS 18 B	4	ALA 49	3.3
ASP 68 I ASP 14 B	1	ASP 71 I ALA 39 B		ARG 87 I ARG 1 B	1		
ASP 71 I ARG 21 B		ASP 71 I ARG 32 B		ASP 48 I ASN 26 B	1		
ASP 71 I ASN 17 B		ASP 71 I ARG 34 B		ASP 65 I ARG 15 B			
ASP 71 I ASP 9 B		ASP 71 I ASN 36 B		ASP 65 I ASN 22 B	2		
ASP 71 I ASP 14 B		ASP 71 I ASP 38 B		ASP 68 I ARG 15 B	1	ASP 68	1
ASP 71 I GLU 16 B		ASP 71 I HIS 35 B		ASP 71 I ARG 15 B			
ASP 71 I GLY 20 B		ASP 71 I LEU 33 B		ASP 71 I ASN 22 B			
ASP 71 I LEU 19 B		ASP 71 I SER 31 B		ASP 71 I ASP 16 B			
ASP 71 I PHE 15 B		ASP 71 I TRP 162 A	9	ASP 71 I LYS 18 B			
ASP 71 I VAL 18 B	9	ASP 89 I TRP 21 B	1	ASP 71 I SER 14 B			
ASP 94 I ARG 21 B		ASP 94 I ASN 36 B		ASP 71 I TYR 23 B			
ASP 94 I ILE 22 B		ASP 94 I ILE 40 B		ASP 71 I VAL 17 B			
ASP 94 I LYS 123 A		ASP 94 I TRP 162 A	3	ASP 71 I VAL 19 B	8	ASP71	8.7
ASP 94 I VAL 18 B	4	CYS 27 I ARG 34 B		ASP 89 I ARG 1 B			
CYS 27 I ASN 17 B	1	CYS 27 I ASP 38 B		ASP 89 I PRO 2 B	2		
CYS 84 I LYS 123 A		CYS 27 I TYR 46 B	3	ASP 94 I TYR 3 B			
GLN 47 I GLU 25 B	1	CYS 50 I ARG 34 B		ASP 94 I TYR 23 B			
GLN 67 I ASN 17 B	1	CYS 50 I ASP 38 B	2	ASP 94 I VAL 19 B	3	ASP 94	3.3
GLU 26 I GLU 16 B	1	GLN 47 I LYS 43 B	1	CYS 27 I LYS 18 B	1	CYS 27	1.7
GLY 70 I ASN 17 B		GLN 67 I ARG 32 B		CYS 78 I ASN 26 B	1		
GLY 70 I ASP 14 B		GLN 67 I HIS 35 B	2	CYS 84 I TYR 23 B	1		
GLY 70 I GLU 16 B		GLU 26 I ARG 34 B		GLN 47 I ASN 26 B			
GLY 70 I VAL 18 B	4	GLU 26 I ASP 38 B		GLN 47 I CYS 25 B	2	GLN 47	1.3
GLY 91 I ASP 122 A		GLU 26 I HIS 35 B		GLN 67 I ARG 32 B			
GLY 91 I CYS 121 A		GLU 26 I TYR 46 B	4	GLN 67 I LYS 18 B	2	GLN 67	1.7
GLY 91 I HIS 120 A		GLY 70 I ARG 32 B		GLU 26 I ALA 21 B			
GLY 91 I LYS 123 A		GLY 70 I ARG 34 B		GLU 26 I LYS 18 B	2	GLU 26	2.3
GLY 91 I SER 4 B	5	GLY 70 I ASN 36 B		GLY 70 I ARG 15 B			
GLY 92 I ARG 21 B		GLY 70 I HIS 35 B		GLY 70 I LYS 18 B			
GLY 92 I ASP 9 B		GLY 70 I LEU 33 B		GLY 70 I SER 14 B			
GLY 92 I ASP 122 A		GLY 70 I SER 31 B	6	GLY 70 I THR 13 B			
GLY 92 I CYS 121 A		GLY 91 I ARG 25 B		GLY 70 I VAL 17 B			
GLY 92 I HIS 120 A		GLY 91 I TRP 21 B	2	GLY 70 I VAL 19 B	6	GLY 70	5.3
GLY 92 I SER 4 B		GLY 92 I ARG 25 B		GLY 91 I ARG 1 B			
GLY 92 I VAL 18 B	7	GLY 92 I TRP 21 B		GLY 91 I GLU 4 B			
SER 66 I ARG 21 B	1	GLY 92 I TRP 162 A		GLY 91 I PRO 2 B			
SER 69 I ASN 17 B		GLY 92 I VAL 158 A	4	GLY 91 I TYR 3 B	4	GLY 91	3.7
SER 69 I ASP 14 B	2	LEU 82 I LYS 43 B	1	GLY 92 I GLU 4 B			
SER 72 I ARG 21 B		LYS 145 I ALA 166 A		GLY 92 I GLU 5 B			
SER 72 I ASN 17 B		LYS 145 I ASP 44 B		GLY 92 I PRO 2 B			
SER 72 I GLY 20 B		LYS 145 I GLU 167 A		GLY 92 I TYR 3 B			
SER 72 I VAL 18 B	4	LYS 145 I TYR 168 A	4	GLY 92 I VAL 19 B	5	GLY 92	5.3
THR 73 I ARG 21 B	1	SER 66 I ALA 39 B		LEU 82 I ASN 26 B	1		
THR 90 I ASP 122 A		SER 66 I ARG 32 B		LYS 145 I GLN 116 A	1		
THR 90 I LYS 123 A	2	SER 66 I ASN 36 B	3	SER 66 I ARG 15 B			
THR 93 I ARG 21 B		SER 69 I ARG 32 B		SER 66 I ASN 22 B			
THR 93 I ASP 9 B		SER 69 I HIS 35 B		SER 66 I VAL 19 B	3	SER 66	2.3
THR 93 I GLU 8 B		SER 69 I SER 31 B	3	SER 69 I LYS 18 B			
THR 93 I HIS 120 A		SER 72 I ALA 39 B		SER 69 I SER 14 B	2	SER 69	2.3
THR 93 I SER 4 B		SER 72 I ARG 34 B		SER 72 I ASN 22 B			
THR 93 I VAL 18 B	6	SER 72 I ASN 36 B		SER 72 I LYS 18 B			
VAL 83 I ILE 22 B		SER 72 I ASP 38 B		SER 72 I VAL 19 B	3	SER 72	4
VAL 83 I LYS 123 A	2	SER 72 I HIS 35 B	5	SER 77 I ASN 26 B	1		
VAL 143 I PHE 131 A	1	THR 73 I ALA 39 B	1	THR 73 I ASN 22 B	1	THR 73	1
		THR 90 I TRP 21 B	1	THR 88 I ARG 1 B	1		
		THR 93 I ARG 25 B		THR 90 I ARG 1 B			
		THR 93 I ARG 32 B		THR 90 I GLU 4 B			
		THR 93 I ASN 36 B		THR 90 I PRO 2 B			
		THR 93 I LEU 28 B		THR 90 I TYR 3 B	4	THR 90	2.3
		THR 93 I TRP 162 A	5	THR 93 I ARG 15 B			
				THR 93 I GLU 4 B			
				THR 93 I TYR 3 B			
				THR 93 I VAL 19 B	4	THR 93	5
				VAL 83 I LEU 27 B			
				VAL 83 I TYR 23 B			

(b)

	IAG - IGFBP_N'	ILP1 - IGFBP_N'	ILP2 - IGFBP_N'
**Number of interacting residues PRODIGY/ PDBsum**			
IGFBP_N'	21/12	21/17	28/14
A Chain	4/4	3/2	1/1
B Chain	15/7	17/11	18/10
**Contact properties PRODIGY**			
charged - charged	9	10	8
charged - polar	12	14	15
charged - apolar	18	21	16
polar - polar	4	4	6
polar - apolar	8	8	13
apolar - apolar	7	7	11

(c)

**Consistent IGFBP_N' contacts**	
**PRODIGY (17)**	**PDBsum (8)**
ALA 49	ILP1
ASP 68	ILP1
**ASP 71**	**All**
**ASP 94**	**All**
CYS 27	ILP1
GLN 47	None
**GLN 67**	**All**
GLU 26	ILP1
**GLY 70**	**All**
**GLY 91**	**All**
**GLY 92**	**All**
SER 66	IAG and ILP2
SER 69	ILP1 and ILP2
**SER 72**	**All**
THR 73	None
THR 90	IAG and ILP2
**THR 93**	**All**
